# Homologous U-box E3 Ubiquitin Ligases OsPUB2 and OsPUB3 Are Involved in the Positive Regulation of Low Temperature Stress Response in Rice (*Oryza sativa* L.)

**DOI:** 10.3389/fpls.2017.00016

**Published:** 2017-01-20

**Authors:** Mi Young Byun, Li Hua Cui, Tae Kyung Oh, Ye-Jin Jung, Andosung Lee, Ki Youl Park, Bin Goo Kang, Woo Taek Kim

**Affiliations:** ^1^Department of Systems Biology, College of Life Science and Biotechnology, Yonsei UniversitySeoul, South Korea; ^2^ReSEAT Program, Korea Institute of Science and Technology InformationSeoul, South Korea

**Keywords:** cold stress, dimerization, E3 Ub ligase, rice (*Oryza sativa*), U-box motif

## Abstract

Rice U-box E3 Ub ligases (OsPUBs) are implicated in biotic stress responses. However, their cellular roles in response to abiotic stress are poorly understood. In this study, we performed functional analyses of two homologous OsPUB2 and OsPUB3 in response to cold stress (4°C). *OsPUB2* was up-regulated by high salinity, drought, and cold, whereas *OsPUB3* was constitutively expressed. A subcellular localization assay revealed that OsPUB2 and OsPUB3 were localized to the exocyst positive organelle (EXPO)-like punctate structures. OsPUB2 was also localized to the nuclei. OsPUB2 and OsPUB3 formed a hetero-dimeric complex as well as homo-dimers in yeast cells and *in vitro*. OsPUB2/OsPUB3 exhibited self-ubiquitination activities *in vitro* and were rapidly degraded in the cell-free extracts with apparent half-lives of 150–160 min. This rapid degradation of OsPUB2/OsPUB3 was delayed in the presence of the crude extracts of cold-treated seedlings (apparent half-lives of 200–280 min). Moreover, a hetero-dimeric form of OsPUB2/OsPUB3 was more stable than the homo-dimers. These results suggested that OsPUB2 and OsPUB3 function coordinately in response to cold stress. *OsPUB2*- and *OsPUB3*-overexpressing transgenic rice plants showed markedly better tolerance to cold stress than did the wild-type plants in terms of survival rates, chlorophyll content, ion leakage, and expression levels of cold stress-inducible marker genes. Taken together, these results suggested that the two homologous rice U-box E3 Ub ligases OsPUB2 and OsPUB3 are positive regulators of the response to cold stress.

## Introduction

Higher plants are constantly subjected to adverse environmental conditions owing to either biotic factors, such as pathogens and herbivores, or abiotic factors, such as extreme temperature, water availability, and high salinity. Being sessile organisms, plants are unable to move to more favorable places, and thus have developed the ability to sense and adjust to stressful conditions. Rice, a monocot model plant and the food source of more than half of the world’s population, is normally grown in tropical and temperate climate zones and is sensitive to chilling stress (Mukhopadhyay et al., 2004). Cold stress affects germination and reduces fertility, which are the key factors responsible for the decline in crop yields (Andaya and Tai, 2006; Suzuki et al., 2008; Ma et al., 2009). Thus, plants with enhanced tolerance to cold stress are able to show better growth (Cabello et al., 2014).

Ubiquitination is a post-translational modification of cellular proteins, which mainly identifies them for degradation via the 26S proteasome complex (Vierstra, 2009; Sadanandom et al., 2012). The ubiquitin (Ub)-proteasome system (UPS) regulates the stability and activity of many proteins and influences diverse cellular processes, including signal transduction, cell division, and response to biotic and abiotic stresses, in higher plants (Santner and Estelle, 2010; Lyzenga and Stone, 2012; Stone, 2014; Zhang et al., 2015; Yu et al., 2016). The UPS is conducted via successive reactions catalyzed by three enzymes (E1 Ub-activating enzymes, E2 Ub-conjugating enzymes, and E3 Ub ligases) that stimulate the tethering of poly-ubiquitin chain to a target protein for its degradation. In general, E3 Ub ligases play a crucial role in the specific recognition of appropriate target proteins and attachment of a poly-ubiquitin chain (Chen and Hellmann, 2013). E3 Ub ligases are divided into two groups based on their structures: single-subunit and multi-subunit E3 ligases (Lee and Kim, 2011; Guerra and Callis, 2012; Sharma et al., 2016). The former group consists of RING (for Really Interesting New Gene)/U-box and HECT (for Homology to E6-AP Carboxyl Terminus) E3 Ub ligases. The latter group includes SCF (for Skp1-Cullin-F-box) and APC (for Anaphase-Promoting Complex) E3 ligases.

The U-box E3 Ub ligases contain a modified RING domain and widely exist in eukaryotic organisms. While yeasts and humans contain 2 and 21 U-box E3 genes, respectively (Koegl et al., 1999; Hatakeyama et al., 2001; Ohi et al., 2003), at least 64 and 77 U-box E3 Ub ligases are predicted to be present in *Arabidopsis* and rice genomes, respectively (Mudgil et al., 2004; Zeng et al., 2008). Increased number of U-box proteins (PUBs) in higher plants might indicate their important roles in the adjustment of diverse cellular processes that are specific to plants (Yee and Goring, 2009). U-box E3 Ub ligases were recently implicated in biotic and abiotic stress responses in higher plants (Trujillo and Shirasu, 2010; Lyzenga and Stone, 2012; Duplan and Rivas, 2014; Stone, 2014; Zhang et al., 2015; Yu et al., 2016).

Rice PUB proteins have been reported to play roles in biotic stress responses. For example, SPL11 is known to ubiquitinate Rho GTPase-activating protein (RhoGAP) SPIN6 and negatively regulate innate immunity in rice (Zeng et al., 2004; Liu et al., 2015). OsPUB15 is involved in reducing cellular oxidative stress during seedling establishment (Park et al., 2011). OsPUB15 interacts with the receptor-like kinase PID2 and regulates cell death and immunity (Wang et al., 2015). OsPUB44 was found to be positively involved in PAMP-triggered immunity (Ishikawa et al., 2014). In addition, rice PUBs are known to participate in various cellular aspects, including brassinosteroid hormone signaling and phosphate starvation response (Hu et al., 2013; Hur et al., 2014; Ren et al., 2014). Nevertheless, the cellular roles of OsPUBs in response to abiotic stress are largely unknown in rice.

In this study, we identified two homologous U-box-type E3 Ub ligases, OsPUB2 and OsPUB3, in rice (*Oryza sativa* L.). The *OsPUB2* gene was up-regulated by low temperature (4°C), whereas the transcript level of *OsPUB3* remained unchanged after 48 h of cold treatment. Subcellular localization assay revealed that OsPUB2 and OsPUB3 were localized to the exocyst positive organelle (EXPO)-like punctate structures that were closely overlapped with Exo70E2 proteins. OsPUB2 was also localized to the nuclei. Yeast-two hybrid and *in vitro* pull-down assays indicated that OsPUB2 and OsPUB3 formed a hetero-dimeric complex as well as homo-dimers. Cell-free protein degradation assay indicated that OsPUB2 and OsPUB3 were more stable when they formed a hetero-dimer than when they formed homo-dimers. Both *OsPUB2*- and *OsPUB3*-overexpressing transgenic rice plants exhibited markedly enhanced tolerance to cold stress compared to wild-type rice plants. These results suggest that OsPUB2 and OsPUB3 are positively involved in the response to cold stress in rice.

## Materials and Methods

### Plants Materials

Rice (*Oryza sativa* L.) japonica variety ‘Dong-jin’ was used in this study. Dry rice seeds were washed with 70% ethanol and subsequently with distilled water. They were then sterilized with 0.4% NaClO solution for 30 min and washed extensively with sterilized water until the NaClO solution was washed off. Sterilized seeds were germinated and grown on half-strength Murashige and Skoog (MS) medium containing vitamins (Duchefa Biochemie, Haarlem, The Netherlands), 3% sucrose, and 0.7% phytoagar for 8–10 days. Seedlings were transplanted to soil and grown at 28°C under long-day (16-h light and 8-h dark) conditions in a green house.

### RNA Extraction, RT-PCR, and Real-Time Quantitative RT-PCR Analyses

Total RNA was extracted from various tissues of wild-type and *Ubi:sGFP-OsPUB2, Ubi:sGFP-OsPUB3, Ubi:RNAi-OsPUB2*, and *Ubi:RNAi-OsPUB3* transgenic rice plants by using Easy Spin Plants Total RNA Extraction kit (iNtRON Biotechnology, South Korea) according to the manufacturer’s protocol. RNA was quantified using a spectrophotometer (NanoDrop1000; Thermo Scientific, USA). Total RNA (2 μg) was used to synthesize cDNA by using TOPscript Reverse Transcriptase (Enzynomics, South Korea) and oligo (dT) primers.

Reverse transcription polymerase chain reaction (RT-PCR) was conducted as described previously (Seo et al., 2012). PCR products were separated on a 2% agarose gel and visualized under UV light. Real-time quantitative RT-PCR (qRT-PCR) was conducted on an IQ5 light cycler (Bio-Rad, USA) in 20 μL reaction mixtures by using SYBR Premix Ex Taq II (TAKARA, Japan). The amplification procedures were as follows: 5 min of denaturation and enzyme activation at 95°C, followed by 50 cycles of 5 s at 95°C, 10 s at 55°C, and 10 s at 72°C. The *Actin* (Os11g06390) gene was used as an internal control, and *DREB1B/CBF1* (Os09g35010; *CCAAT-binding factor*) was used as a positive control for cold stress treatment. *GAD* (Os03g13300; *glutamate decarboxylase*), *WRKY77* (Os01g40260), *MRP4* (Os01g50100; *multidrug resistance protein 4*), *MYBS3* (Os01g50100), *TPP2* (Os10g40550; *trehalose-6-phosphate phosphatase* 2), and *DREB1B/CBF1* were cold stress-induced genes (Jain et al., 2007; Su et al., 2010). Gene-specific primers used for PCR are listed in Supplementary Table S1.

### *In vitro* Self-Ubiquitination Assay

*In vitro* self-ubiquitination assay was performed according to the established protocol described in a previous study (Bae et al., 2011). The *Myc-OsPUB2, Myc-OsPUB3, Myc-OsPUB2C281A*, and *Myc-OsPUB3C280A* fusion genes were cloned to pProEx hta vectors (Invitrogen, USA). Briefly, bacterially expressed Myc-OsPUB2, Myc-OsPUB2C281A, Myc-OsPUB3, and Myc-OsPUB3C280A recombinant fusion proteins (500 ng) were incubated for 2 h in the presence or absence of 100 ng E1 (*Arabidopsis* UBA1), 100 ng E2 (*Arabidopsis* UBC8), 10 mM ATP, and 0.1 μg/mL Ub with ubiquitination reaction buffer (50 mM Tris-HCl, pH 7.5, 2.5 mM MgCl_2_, and 0.5 mM DTT) at 30°C. The reaction products were subjected to immuno-blotting by using anti-Myc (Applied Biological Materials, Canada) and anti-Ub (Santa Cruz Biotechnology, USA) antibodies.

### Subcellular Localization

The 3′ ends of *OsPUB2* and *OsPUB3* coding regions were tagged with synthetic green fluorescent protein (*sGFP*) or monomeric red fluorescent protein (*mRFP*) in-frame and inserted into pEarleyGate (pEG) 100 binary vectors that contains the 35S CaMV promoter. The vector was then transformed into *Agrobacterium tumefaciens* strain LBA4404 by electroporation. The *35S:OsPUB2-sGFP, 35S:OsPUB2-mRFP, 35S:OsPUB3-sGFP, 35S:OsPUB3-mRFP, 35S:NLS-mRFP*, and *35S:AtExo70E2-sGFP* constructs were expressed in tobacco (*Nicotiana benthamiana*) leaves by using *Agrobacterium-*mediated method as described in a previous study (Kim and Kim, 2013). Two days after infection, protoplasts were extracted from the tobacco leaves, and fluorescent protein signals were visualized by fluorescence microscopy (BX51, Olympus, Japan) as described by Byun et al. (2015). NLS-mRFP and AtExo70E2-sGFP were used as nucleus and EXPO marker proteins, respectively.

Rice protoplasts were obtained from 11-day-old seedlings of *Ubi:sGFP-OsPUB2* and *Ubi:sGFP-OsPUB3* transgenic rice plants before and after 48 h cold treatment and were used for subcellular localization analysis of OsPUB2 and OsPUB3. Fluorescent signals were visualized by fluorescence microscopy in the presence of 50 μM MG132.

### Generation of Transgenic Rice Plants

Transgenic rice plants were produced by transforming the pGA2897 binary vector plasmids that contained the maize ubiquitin promoter (*Ubi*) and *OsPUB2/OsPUB3* (*Ubi:sGFP-OsPUB2, Ubi:sGFP-OsPUB3, Ubi:RNAi-OsPUB2*, and *Ubi:RNAi-OsPUB3*) into the *A. tumefaciens* strain LBA4404 via electroporation. Callus was generated by germinating wild-type rice (*Oryza sativa* L. japonica variety called ‘Dong-Jin’) seeds on the callus induction medium (2 mg L^-1^ 2,4-D, 0.003% casein hydrolysate, 0.4% CHU stock containing vitamins [Duchefa Biochemie], 0.2% gelite, 0.03% L-proline, and 3% sucrose, pH 5.8) and used for *Agrobacterium*-mediated rice transformation. Transformed callus was selected on hygromycin B (40 mg L^-1^) and carbenicillin (250 mg L^-1^) containing medium and then transferred to the regeneration medium (2 mg L^-1^ kinetin, 4% MS medium containing B5 vitamins, 1 mg L^-1^ NAA, 1.2% phytoagar, 2% sorbitol, and 5% sucrose, pH 5.8). All processes during rice transformation were followed as described previously (Byun and Kim, 2014). Transgenic T0 plants were transplanted to soil and independent T4 overexpressing (lines #1 and #2) and T3 RNAi knock-down (lines #1 and #2) transgenic rice plants were used for phenotypic analysis.

### Genomic DNA Extraction and DNA Gel Blot Analysis

Total genomic DNA was extracted from developing leaves of wild-type and transgenic rice plants by using the CTAB (2% CTAB, 2% PVP-40, 1.4 M NaCl, 100 mM Tris-HCl, pH 8.0, and 20 mM EDTA) method as described by Byun and Kim (2014). Total genomic DNA (10 μg) was digested with *Bam*HI or *Eco*RI restriction enzyme (Thermo Scientific, USA) and separated by electrophoresis on 0.7% agarose gel. Separated DNA on the gel was transferred to Hybond-N nylon membrane, and the blot was hybridized with 32P-labeled hygromycin B phosphotransferase (*Hph*) probes under high stringency conditions (65°C) as described by Byun and Kim (2014). The autoradiography signals were visualized using the Bio-Imaging Analyzer (BAS2500; Fuji Film, Japan).

### Yeast Two-Hybrid Assays

Yeast two-hybrid assays were performed according to the method of Bae and Kim (2013) with modifications. The full-length *OsPUB2* and *OsPUB3* coding regions were inserted into pGAD T7 and pGBK T7 vectors (Clontech, USA), respectively. These constructs or empty vector were co-transformed into AH109 yeast cells by using the LiAc/single-stranded carrier DNA/PEG method. Transformed yeast cells were serially diluted, plated onto four-minus (-Leu/-Trp/-His/-Ade) medium and grown at 30°C for 2 days (Bae and Kim, 2014).

### *In vitro* Pull-Down Assays

OsPUB2 and OsPUB3 were cloned into pProEx hta vector and pMAL c2X vector (New England BioLabs, UK) plasmids, respectively. Maltose binding protein (MBP) and (His)_6_-OsPUB2, (His)_6_-OsPUB3, MBP-OsPUB2, and MBP-OsPUB3 recombinant proteins were expressed in *Escherichia coli* BL21 (DE3). Expressed proteins were purified by affinity chromatography by using Ni-NTA agarose (Qiagen, Germany) for (His)_6_-tagged proteins and MBP Excellose (TAKARA, Japan) for MBP fused proteins, respectively. The recombinant proteins were co-incubated with His resin for 3 h at 4°C in the affinity precipitation (AP) buffer (50 mM Tris-HCl, pH 7.5, 1 mM EDTA, 1 mM MgCl_2_, 0.5× proteinase inhibitor cocktail, and 0.3% Triton X-100) and captured proteins were washed five times with AP buffer. The bound proteins were eluted, resolved on 10% SDS-PAGE, transferred to PVDF membrane (Millipore, Germany), and subjected to immuno-blot analysis by using anti-MBP antibody (Applied Biological Materials) or anti-His antibody (Applied Biological Materials).

### Cell-Free Degradation Assays

Cell-free degradation assays were conducted as described by Kim and Kim (2013). Cell-free protein crude extracts were prepared from 8-day-old mock or cold-treated rice seedlings by using protein extraction buffer (50 mM Tris-HCl, pH 7.2, 100 mM NaCl, and protease inhibitor cocktail [Roche, Switzerland]). *Arabidopsis RGA*, which was tagged with *Flag*, was cloned into pProEx hta vector. Bacterially expressed MBP-OsPUB2, MBP-OsPUB3, and Flag-RGA recombinant proteins were incubated with cell-free extracts for 2, 4, and 6 h at 30°C. Each sample was harvested, boiled, separated by 10% SDS-PAGE, and analyzed by immunoblotting by using the anti-MBP antibody and anti-Flag antibody (Applied Biological Materials). Rubisco was used as a loading control.

### Phenotypic Analysis of Wild-Type and Transgenic Rice Plants in Response to Cold Stress

For cold stress treatment, 5-week-old wild-type and transgenic rice plants were transferred to cold room at 4°C for 6 days, after which plants were recovered at 28°C and their growth patterns were monitored as described previously (Byun et al., 2015). Total chlorophyll (chlorophyll a + chlorophyll b) was extracted from wild-type and transgenic leaves before and after cold treatment according to Lichtenthaler (1987) with modifications as described by Min et al. (2016). The amounts of chlorophyll a and chlorophyll b were measured at 664.2 and 648.6 nm, respectively, by ELISA microplate reader (VERSAmax, Molecular Devices, USA).

Electrolyte leakage analysis was conducted using 8-day-old rice seedlings before and after cold stress treatments in different time points (0, 6, and 11 days) at 4°C. Seedlings of wild-type and transgenic plants were soaked in a test tube containing 35 mL of distilled water on an orbital shaker (200 rpm) at 28°C for overnight. The electrolyte conductivity of each sample was determined before and after autoclaving by using conductivity meter (Orion Star A212, Thermo Scientific, USA) by the method of Min et al. (2016).

## Results

### OsPUB2 and OsPUB3 are U-box E3 Ub Ligases in Rice

**FIGURE 1:**
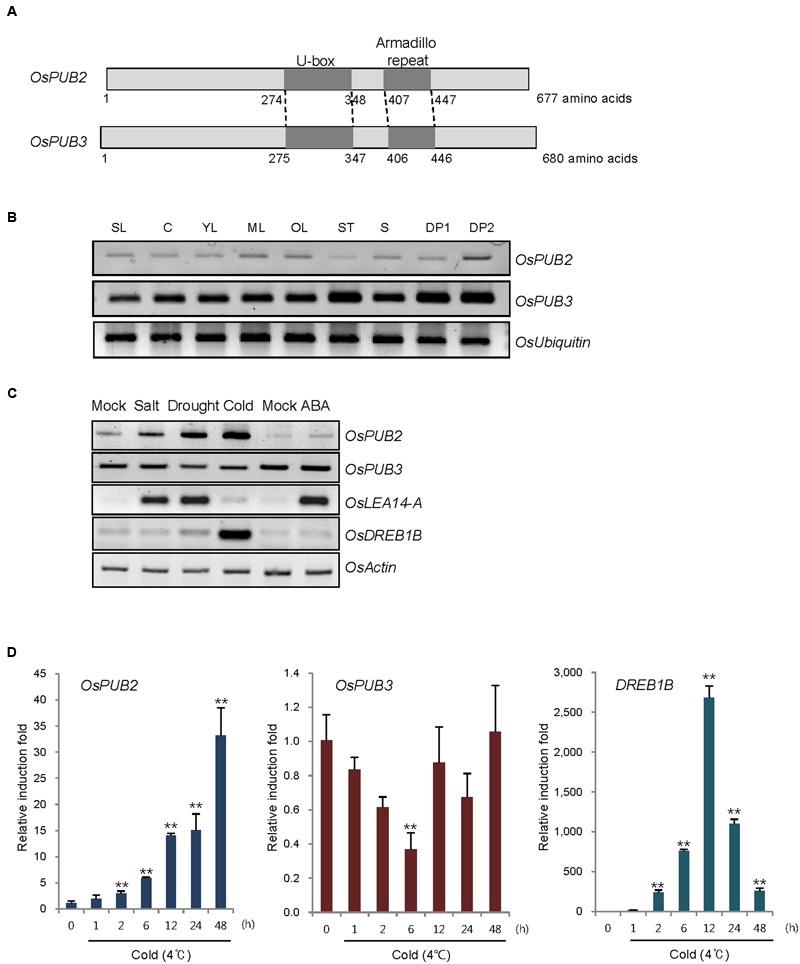
**Identification and expression of *OsPUB2* and *OsPUB3* in rice. (A)** Schematic structures of predicted rice U-box E3 Ub ligases OsPUB2 and OsPUB3. Gray bars depict the coding regions. The U-box motif and Armadillo repeat are indicated by dark gray bars. **(B)** Spatial expression patterns of *OsPUB2* and *OsPUB3* in rice. Total RNA was extracted from various tissues and analyzed by RT-PCR. *OsUbiquitin* is a loading control. SL, 10-day-old whole seedlings; C, callus; YL, young leaves; ML, mature leaves; OL, old leaves; ST, stems; S, seeds; DP1, 3–5 cm-long developing panicles; DP2, 5–8 cm-long developing panicles. **(C)** (Left panel) Expression profiles of *OsPUB2* and *OsPUB3* in response to the different abiotic stress conditions. Light-grown, 10-day-old wild-type rice seedlings were subjected to high salt (200 mM NaCl for 3 h), dry (40% reduction of fresh weight), low temperature (4°C for 8 h), and ABA (150 μM for 3 h). Total RNA was prepared from the treated tissues and analyzed by RT-PCR using gene-specific primer sets. *OsLEA14-A* served as a positive control for drought, salt, and ABA treatments, whereas *OsDREB1B* was used as a cold stress marker gene. *OsActin* is a loading control. (Right panel) Time course inductions of *OsPUB2* and *OsPUB3* in response to cold (4°C for 4 and 8 h) and drought (15, 30, and 45% reduction of fresh weight) treatments. **(D)** Real-time qRT-PCR analysis of *OsPUB2* and *OsPUB3*. Total RNA was isolated from the leaves treated with cold stress (4°C) for different time periods (0, 1, 2, 6, 12, 24, and 48 h) and used for qRT-PCR. Fold induction of *OsPUB2* and *OsPUB3* was normalized to the level of *OsActin* mRNA, which was used as an internal control. Data represent mean ± SE (^∗∗^*P* < 0.01, Student’s *t*-test) from three independent experiments. Nucleotide sequences of primers used for RT-PCR are shown in Supplementary Table S1.

*OsPUB2* and *OsPUB3* are homologous genes that encode putative U-box E3 Ub ligases with 75% amino-acid sequence identity in rice (**Figure 1A**). Both predicted proteins contained a single U-box motif and Armadilo (ARM) repeats in their central and C-terminal regions, respectively. OsPUB2 and OsPUB3 are 71–82% identical to putative U-box proteins from monocot plants, such as millet (*Setaria italica*), false brome (*Brachypodium distachyon*), and maize (*Zea mays*), whereas they share 42% sequence identity with AtPUB17 from dicot *Arabidopsis thaliana* (Supplementary Figure S1). The U-box and ARM domains are well-conserved in both monocot and dicot U-box proteins.

Reverse transcription polymerase chain reaction analysis showed that transcripts of *OsPUB2* and *OsPUB3* were detected in all tissues examined, including early seedlings, developing and mature leaves, stems, developing seeds, and panicles, with the expression level of *OsPUB3* being higher than that of *OsPUB2* (**Figure 1B**). The transcript level of *OsPUB2* was elevated in response to high salt (200 mM NaCl for 3 h), dry (15–45% reduction of fresh weight), and low temperature (4°C for 4–8 h) treatments in 10-day-old rice seedlings (**Figure 1C**). In contrast, the transcript level of *OsPUB3* was not affected by stress treatments. Neither of the genes was induced by ABA (150 μM for 3 h). Real-time qRT-PCR assay indicated that the amount of *OsPUB2* mRNA began to increase at 1 h after exposure to cold stress and was continuously elevated up to 35-fold after 48 h (**Figure 1D**). The induction pattern of *OsPUB2* was different from that of *DREB1B*, a cold stress marker gene, the expression of which was maximum at 12 h after cold treatment. The transcript level of *OsPUB3* was reduced at 6 h after cold treatment and restored to normal levels thereafter.

**FIGURE 2:**
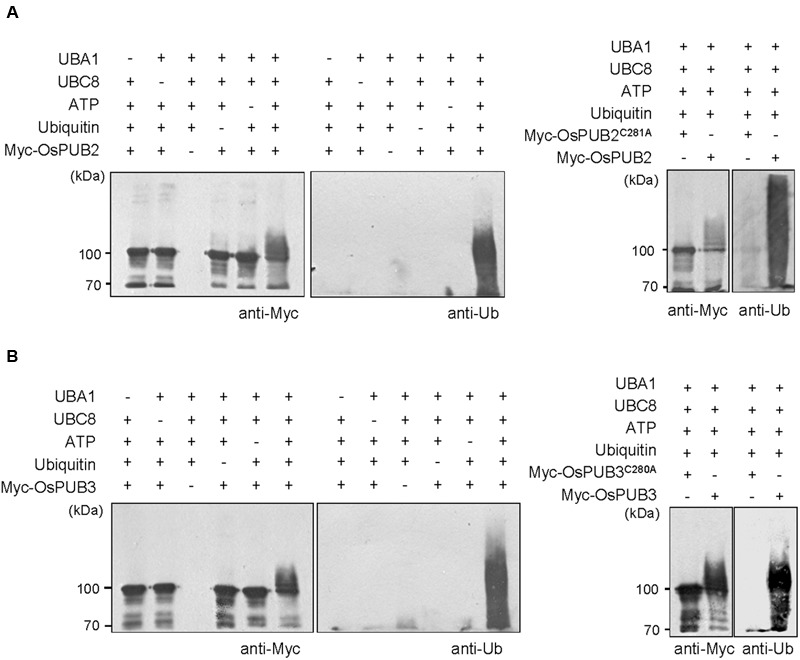
***In vitro* self-ubiquitination assays of OsPUB2 and OsPUB3. (A)** (Left panel) Bacterially expressed Myc-tagged OsPUB2 fusion protein was purified and incubated at 30°C for 2 h in the presence or absence of E1 (*Arabidopsis* UBA1), E2 (*Arabidopsis* UBC8), Ub, and ATP. Samples were resolved by 8% SDS-PAGE and subjected to immuno-blot analysis by using anti-Myc and anti-Ub antibodies. (Right panel) Wild-type Myc-OsPUB2 and a single amino acid substitution mutant Myc-OsPUB2^C281A^ were incubated at 30°C for 2 h with E1, E2, Ub, and ATP, and analyzed by immuno-blotting by using anti-Myc and anti-Ub antibodies. **(B)** Purified recombinant Myc-OsPUB3 (Left panel) and a single amino acid substitution mutant Myc-OsPUB3^C280A^ (Right panel) proteins were used for *in vitro* self-ubiquitination assay by using anti-Myc and anti-Ub antibodies as described above.

Whether OsPUB2 and OsPUB3 possess E3 Ub ligase enzymatic activity was determined by performing *in vitro* self-ubiquitination assay. Bacterially expressed Myc-tagged OsPUB2 or OsPUB3 recombinant proteins were incubated at 30°C for 2 h in the presence or absence of E1(*Arabidopsis* UBA1), E2 (*Arabidopsis* UBC8), Ub, and ATP. Reaction mixtures were separated by SDS-PAGE and subjected to immuno-blot analysis by using anti-Myc and anti-Ub antibodies. As shown in **Figure 2**, Myc-OsPUB2 and Myc-OsPUB3 yielded high-molecular-mass ubiquitinated bands detected by both anti-Myc and anti-Ub antibodies. In contrast, the exclusion of E1, E2, ATP, or Ub from the incubation mixture abrogated ubiquitinated smear bands. Myc-OsPUB2^C281A^ and Myc-OsPUB3C^280A^, in which the conserved Cys residue in the U-box motif was replaced by Ala, failed to exhibit the E3 ligase activity even in the presence of all reaction components (**Figure 2**). Overall, the results presented in **Figures 1** and **2** indicated that OsPUB2 and OsPUB3 are homologous U-box E3 Ub ligases in rice.

### Subcellular Localization of OsPUB2 and OsPUB3

**FIGURE 3:**
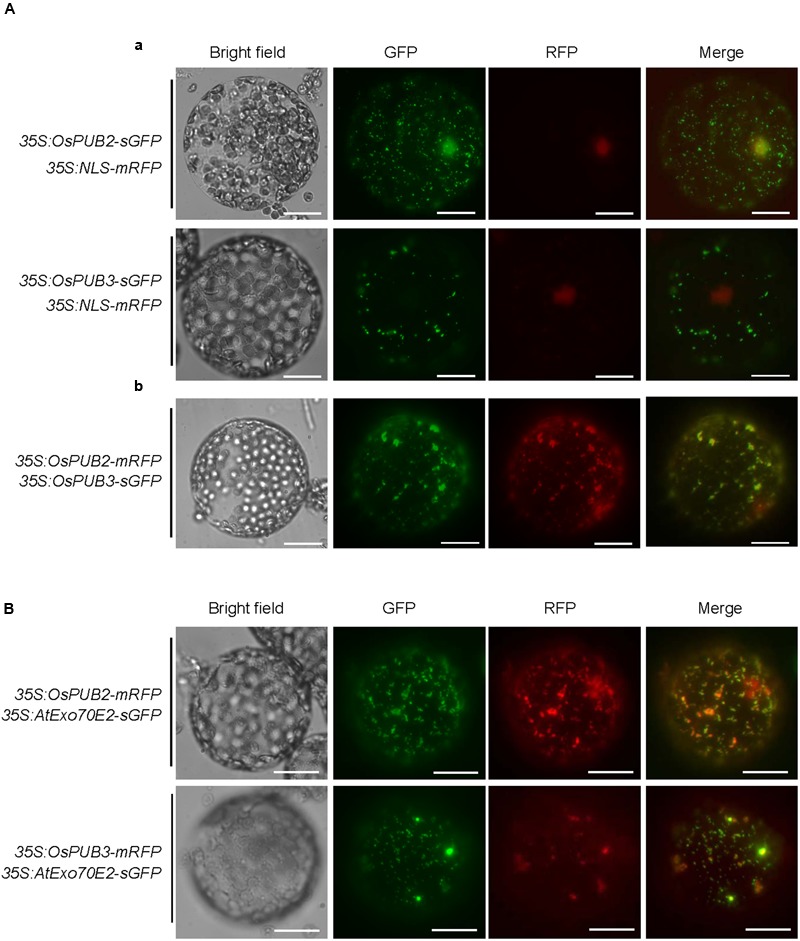
**Subcellular localization of OsPUB2 and OsPUB3. (A-a**) The *35S:OsPUB2-sGFP* or *35S:OsPUB3-sGFP* fusion construct was co-expressed with *35S:NLS-mRFP* in tobacco (*N. benthamiana*) leaf epidermal cells by using *Agrobacterium*-mediated infiltration method. After 2 days of infiltration, protoplasts were prepared from the leaves, and fluorescent signals were visualized by fluorescence microscopy under dark-field conditions. The NLS-mRFP was used as a nucleus-localized marker protein. Bars = 10 μm. **(A-b)** The *35S:OsPUB2-mRFP* and *35S:OsPUB3-sGFP* fusion constructs were co-expressed in tobacco leaves. The co-localization signals of OsPUB2-mRFP and OsPUB3-sGFP in the protoplasts were detected by fluorescence microscopy under dark-field conditions. Bars = 10 μm. **(B)** The *35S:OsPUB2-mRFP*+ *35S:AtExo70E2-sGFP* and *35S:OsPUB3-mRFP*+ *35S:AtExo70E2-sGFP* constructs were infiltrated into tobacco leaves, and their co-localization signals were visualized by fluorescence microscopy. The AtExo70E2-sGFP served as a marker protein of exocyst positive organelles (EXPOs). Bars = 10 μm.

The subcellular localization of OsPUB2 and OsPUB3 was investigated by conducting an *in vivo* protein targeting experiment. The *35S:OsPUB2-sGFP* or *35S:OsPUB3-sGFP* chimeric construct was co-expressed with *35S:NLS-mRFP* in tobacco (*N. benthamiana*) leaves by using *Agrobacterium*-mediated infiltration method. The NLS-mRFP was used as a nuclear marker protein. Protoplasts were prepared from tobacco leaves, and expressed fusion proteins were visualized by fluorescence microscopy. The results revealed that the fluorescence signal of OsPUB2-sGFP was exhibited as small cytosolic punctate bodies and was also found in the nucleus, where it merged with the NLS-mRFP signal (**Figure 3Aa**). In the case of OsPUB3-sGFP, the fluorescence signals were only exhibited in the cytosolic punctae. When *OsPUB2-mRFP* and *OsPUB3-sGFP* constructs were co-expressed in tobacco leaves, the cytosolic punctate fluorescence signals were closely merged, indicating the co-localization of OsPUB2 and OsPUB3 (**Figure 3Ab**). These punctate localization patterns of OsPUB2/OsPUB3 were reminiscent of EXPOs (Wang et al., 2010; Ding et al., 2014). Indeed, the localization signals of OsPUB2-mRFP and OsPUB3-mRFP closely overlapped with those of AtExo70E2-sGFP, a marker protein of EXPO, in tobacco leaf protoplasts (**Figure 3B**). We detected very weak punctate localization signals of both OsPUB2 and OsPUB3 in the protoplasts prepared from *Ubi:OsPUB2-sGFP* and *Ubi:OsPUB3-sGFP* transgenic rice plants (Supplementary Figure S3). These localization patterns were unchanged in response to cold treatment (Supplementary Figure S3). Thus, OsPUB2 appeared to be localized to the EXPO-like punctate bodies and nuclei, whereas OsPUB3 was predominantly localized to the EXPO-like structure.

### OsPUB2 and OsPUB3 Formed Homo- and Hetero-Dimeric Complexes

**FIGURE 4:**
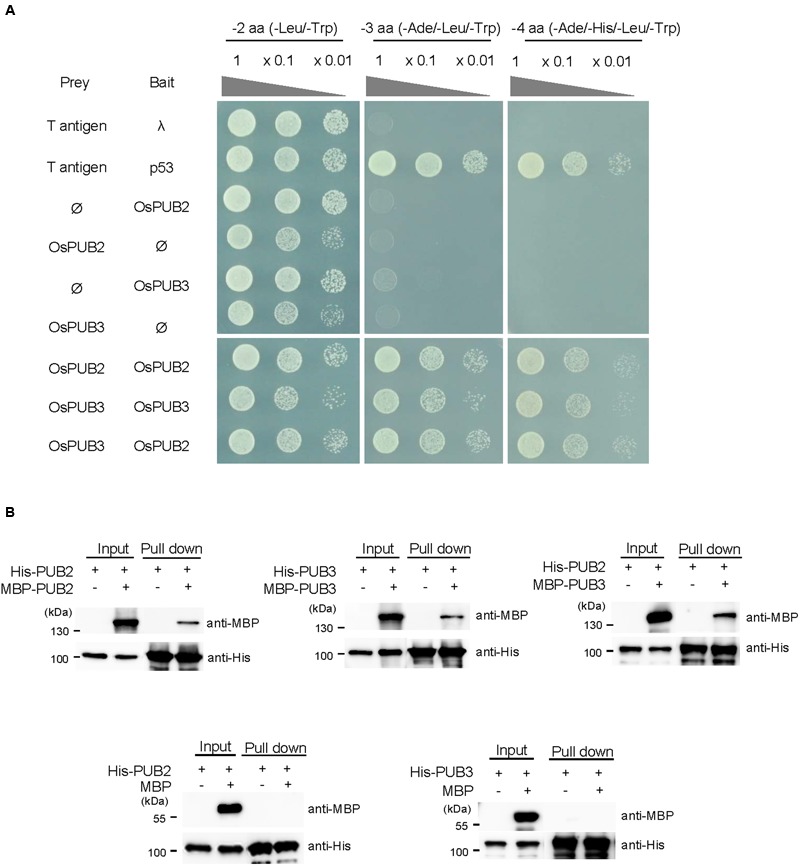
**Homo- and hetero-dimeric complex formation of OsPUB2 and OsPUB3. (A)** Yeast two-hybrid assay. The full-length coding regions of OsPUB2 and OsPUB3 were cloned into pGAD T7 and pGBK T7 vectors, respectively, and co-transformed into yeast AH109 cells with a combination of the indicated plasmids. Transformed yeast cells were serially diluted, plated onto two-minus (-Leu/-Trp), three-minus (-Leu/-Trp/-Ade), and four-minus (-Leu/-Trp/-His/-Ade) growth media, and incubated at 30°C for 2 days. The p53 + T-antigen were used as a positive control, whereas lambda + T-antigen were used as a negative control. **(B)** *In vitro* pull down assay. Bacterially expressed MBP-OsPUB2, MBP-OsPUB3, or MBP recombinant protein was co-incubated with (His)_6_-OsPUB2 or (His)_6_-OsPUB3 as indicated in the presence of a Ni-NTA agarose affinity matrix. The bound protein was eluted, resolved by 10% SDS-PAGE, and immuno-blotted with anti-MBP and anti-His antibodies.

The RING/U-box E3 Ub ligases often form stable dimeric complexes, which is critical for their activity (Nikolay et al., 2004; Berndsen and Wolberger, 2014). To determine whether OsPUB2 and OsPUB3 form dimers, we performed a yeast two-hybrid assay. The full-length *OsPUB2* and *OsPUB3* coding regions were inserted into pGAD T7 and pGBK T7 vectors, respectively, and co-transformed into yeast cells. Transformed yeast cells were serially diluted and plated onto three-minus (-Leu/-Trp/-Ade) and four-minus (-Leu/-Trp/-His/-Ade) growth media. The results showed that both OsPUB2 and OsPUB3 could interact with themselves and formed homo-dimers in the yeast cells (**Figure 4A**). Considering the high sequence identity (75%) (**Figure 1A**; Supplementary Figure S1A) and identical subcellular localization patterns (**Figure 3**) of OsPUB2 and OsPUB3, we speculated that they formed a hetero-dimeric complex. As expected, OsPUB2 and OsPUB3 bound each other to form hetero-dimers in the yeast cells (**Figure 4A**).

To further confirm the dimeric-complex formation of OsPUB2 and OsPUB3, we performed *in vitro* pull-down assay. Bacterially expressed (His)_6_-OsPUB2 + MBP-OsPUB2, (His)_6_-OsPUB3 + MBP-OsPUB3, and (His)_6_-OsPUB2 + MBP-OsPUB3 protein mixtures were co-incubated with a Ni-NTA resin. The bound proteins were eluted from the resin and analyzed by immuno-blotting by using anti-MBP and anti-His antibodies. As indicated in **Figure 4B**, the MBP-tagged OsPUB2 and OsPUB3 proteins were pull-downed from the Ni-NTA resin by the (His)_6_-tagged OsPUB2 and OsPUB3, indicating that OsPUB2 and OsPUB3 formed both homo- and hetero-dimeric complexes *in vitro*.

### OsPUB2 and OsPUB3 are More Stable in Cell-Free Extracts When They Form a Hetero-Dimer

**FIGURE 5:**
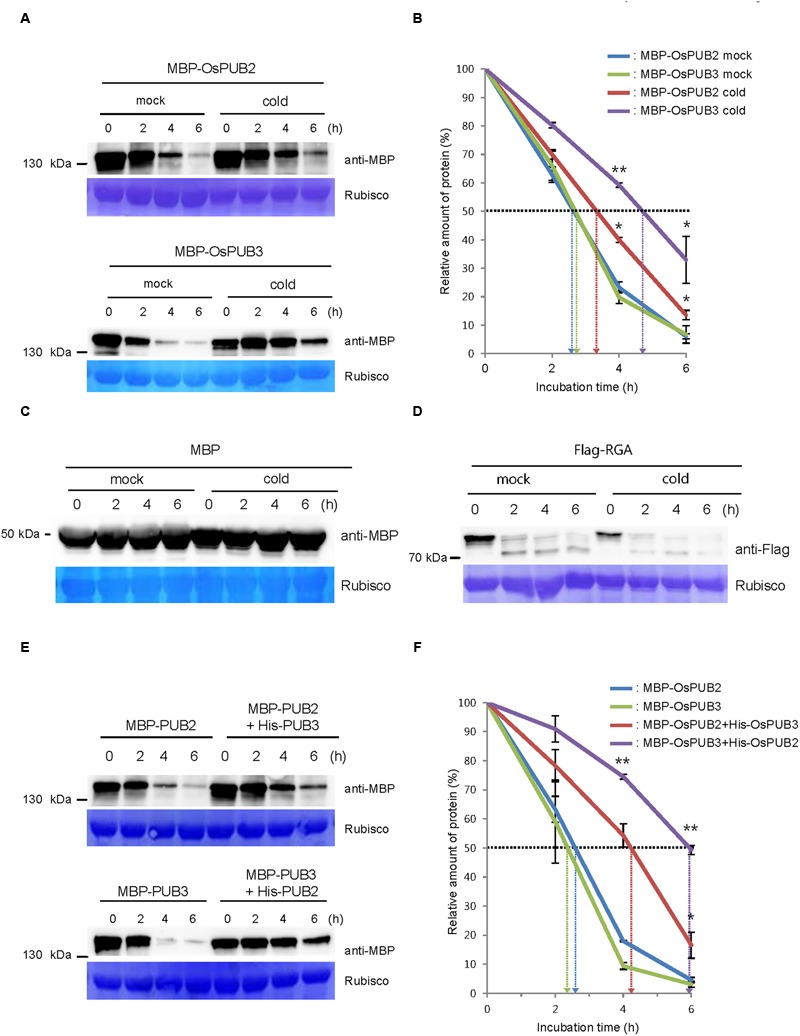
**Regulation of OsPUB2 and OsPUB3 stability in cell-free protein crude extracts. (A)** *In vitro* cell-free degradation assays of OsPUB2 and OsPUB3 under mock and cold stress conditions. The MBP-OsPUB2 or MBP-OsPUB3 protein was incubated for different time periods (0, 2, 4, and 6 h) with the protein crude extracts prepared from light-grown 8-day-old wild-type rice seedlings before and after cold treatment. The time-dependent protein levels were analyzed by immuno-blotting by using anti-MBP antibody. Rubisco detected by Coomassie blue served as a loading control. **(B)** Apparent half-lives of OsPUB2 and OsPUB3 in the cell-free crude extracts. The graph shows the decrease in the relative amounts of proteins in the cell-free degradation assay. The levels of proteins were calculated by quantification of band intensities by using ImageJ software. Bars represent mean ± SE (^∗^*P* < 0.05, ^∗∗^*P* < 0.01, Student’s *t*-test) from three independent experiments. **(C,D)** *In vitro* cell-free degradation assays of MBP and RGA under mock and cold stress conditions. The MBP **(C)** or Flag-RGA **(D)** protein was incubated for different time periods (0, 2, 4, and 6 h) with the protein crude extracts prepared from light-grown 8-day-old wild-type rice seedlings before and after cold treatment. The time-dependent protein levels were analyzed by immuno-blotting by using anti-MBP **(C)** and anti-Flag **(D)** antibodies. Rubisco detected by Coomassie blue served as a loading control. **(E)** *In vitro* cell-free degradation assay of homo- and hetero-dimeric complexes of OsPUB2 and OsPUB3. The MBP-OsPUB2 and MBP-OsPUB3 were incubated for different time periods (0, 2, 4, and 6 h) in the presence or absence of (His)_6_-OsPUB3 and (His)_6_-OsPUB2, respectively, with mock-treated crude extracts. The levels of proteins were detected by immuno-blotting with anti-MBP antibody. Rubisco was used as a loading control. **(F)** Apparent half-lives of OsPUB2 and OsPUB3 in the cell-free crude extracts when they formed homo- and hetero-dimers. The graph shows the decrease in the relative amounts of proteins in the cell-free degradation assay. Bars indicate mean ± SE (^∗^*P* < 0.05, ^∗∗^*P* < 0.01, Student’s *t*-test) from three independent experiments.

We next considered the possibility that the formation of a hetero-dimeric complex of OsPUB2 and OsPUB3 might affect their stability. To test this hypothesis, we performed an *in vitro* cell-free degradation assay. MBP-OsPUB2 and MBP-OsPUB3 recombinant proteins were incubated for different time periods (0, 2, 4, and 6 h) with the crude protein extracts prepared from 8-day-old rice seedlings grown under normal conditions. The levels of MBP-OsPUB2 and MBP-OsPUB3 rapidly decreased over time (0–6 h) in the cell-free extracts. At 4 h of incubation, 76.8 ± 2.0 and 80.2 ± 2.2% of OsPUB2 and OsPUB3 were degraded, respectively (**Figure 5A**). After 6 h, only 6.0 ± 0.9 and 6.7 ± 3.0% of OsPUB2 and OsPUB3 proteins were detected, respectively. Under our experimental conditions, the apparent half-lives of MBP-OsPUB2 and MBP-OsPUB3 were approximately 150 min and 160 min, respectively, in the mock-treated cell-free extracts (**Figure 5B**). However, the degradation of MBP-OsPUB2 and MBP-OsPUB3 appeared to be slower in the cell-free extracts prepared from the cold-treated seedlings than in the mock-treated seedlings: apparent half-lives of MBP-OsPUB2 and MBP-OsPUB3 were about 200 min and 280 min, respectively, in the cold-treated cell-free extracts (**Figure 5B**). We repeated identical sets of experiments using MBP as a negative control. As shown in **Figure 5C**, the level of MBP was consistent in mock- and cold-treated crude extracts. In addition, RGA, which was degraded by the 26S proteasome complex (Lee et al., 2010; Kim and Kim, 2013), was degraded in a cold-independent manner (**Figure 5D**). These results suggested that OsPUB2 and OsPUB3 are more stable in cold-treated cell-free extracts than in mock-treated extracts, with the half-life of OsPUB3 being more apparently increased.

Furthermore, when OsPUB2 and OsPUB3 were mixed together in the cell-free extracts, their degradation was markedly delayed (**Figure 5E**); thus, their apparent half-lives were increased from 150 min and 140 min to 250 min and 350 min, respectively (**Figure 5F**). On the other hand, both OsPUB2 and OsPUB3 were rapidly degraded in the presence of equal amount of RGA (Supplementary Figures S2A,B), suggesting that slower degradation of OsPUB2 + OsPUB3 hetero-dimer was not due to the increased amounts of proteins in the reaction mixture. This indicated that a hetero-dimeric form of OsPUB2 and OsPUB3 was more stable than the homo-dimers in a cell-free system. Taken together, these results raised the possibility that the stability of OsPUB2 and OsPUB3 are ameliorated in response to cold stress (**Figures 5A,B**) and by the formation of a hetero-dimeric complex (**Figures 5E,F**).

### Generation and Molecular Analysis of *OsPUB2*- and *OsPUB3*-Overexpressing and RNAi-Mediated Knock-Down Transgenic Rice Plants

**FIGURE 6:**
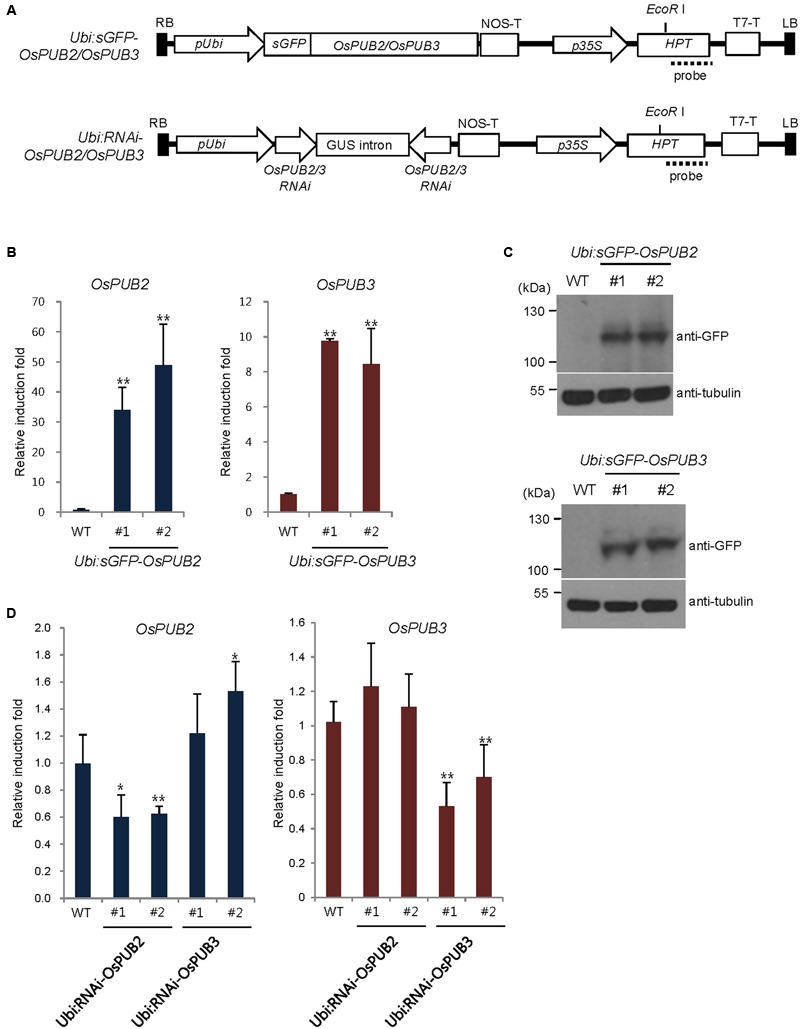
**Generation and molecular characterization of *OsPUB2*- and *OsPUB3*-overexpressing and *RNAi*-mediated knock-down transgenic rice plants. (A)** Schematic structures of the *OsPUB2* and *OsPUB3* overexpression and *RNAi*-mediated knock-down binary vector constructs. RB, right border; *pUbi*, maize ubiquitin promoter; NOS-T, NOS terminator; *p35S*, 35S CaMV promoter; *Hpt*, hygromycin phosphotransferase; T7-T, T7 terminator; LB, left border. Dash lines indicate DNA probes for genomic Southern blot analysis in Supplementary Figure S4. **(B)** qRT-PCR analysis of wild-type (WT) and T4 *Ubi:sGFP-OsPUB2* (independent transgenic lines #1 and #2) and *Ubi:sGFP-OsPUB3* (lines #1 and #2) transgenic rice plants. Data represent the fold induction of *OsPUB2* and *OsPUB3* in overexpressing transgenic rice plants relative to wild-type plants. The relative expression levels of *OsPUB2* and *OsPUB3* transcripts were normalized to the level of *OsActin* mRNA, which was used as an internal reference gene. Data represent mean ± SE (^∗∗^*P* < 0.01, Student’s *t*-test) from three independent experiments. **(C)** Immuno-blot analysis of wild-type (WT) and T4 *Ubi:sGFP-OsPUB2* (independent transgenic lines #1 and #2) and *Ubi:sGFP-OsPUB3* (lines #1 and #2) transgenic rice plants by using anti-GFP antibody. Tubulin was used as the loading control. **(D)** qRT-PCR analysis of wild-type (WT) and T3 *Ubi:RNAi-OsPUB2* (independent transgenic lines #1 and #2), and *Ubi:RNAi-OsPUB3* (lines #1 and #2) transgenic rice plants. Data represent the fold reduction of *OsPUB2* and *OsPUB3* in *RNAi-*knock-down transgenic progeny relative to wild-type (WT) plants. The relative expression levels of *OsPUB2* and *OsPUB3* mRNAs were normalized to that of *OsActin* mRNA, an internal control. Data represent mean ± SE (^∗^*P* < 0.05, ^∗∗^*P* < 0.01, Student’s *t*-test) from three independent experiments.

To address the cellular roles of OsPUB2 and OsPUB3, we generated transgenic rice plants (*Ubi:sGFP-OsPUB2* and *Ubi:sGFP-OsPUB3*), in which the *sGFP-OsPUB2* and *sGFP-OsPUB3* genes were ectopically expressed under the control of the maize ubiquitin promoter (*Ubi*) (**Figure 6A**). The qRT-PCR analysis results showed markedly increased amounts of *OsPUB2* and *OsPUB3* transcripts in two different T4 transgenic lines (#1 and #2) of *Ubi:sGFP-OsPUB2* and *Ubi:sGFP-OsPUB3* plants, respectively, under normal growth conditions (**Figure 6B**). The overexpression of sGFP-OsPUB2 and sGFP-OsPUB3 proteins was confirmed by immuno-blot analysis by using anti-GFP antibody (**Figure 6C**). DNA gel-blot analysis showed that these over-expressing transgenic lines were independent (Supplementary Figure S4). In addition, the independent transgenic rice plants (*Ubi:RNAi-OsPUB2* and *Ubi:RNAi-OsPUB3*), in which *OsPUB2* and *OsPUB3* were suppressed, were constructed using the RNA interference (RNAi) method (**Figure 6A**; Supplementary Figure S4). The results of qRT-PCR analysis indicated that the expression of *OsPUB2* was reduced approximately by 40% in *Ubi:RNAi-OsPUB2* (transgenic lines #1 and #2), whereas that of *OsPUB3* was decreased by 50% (line #1) to 70% (line #2) in *Ubi:RNAi-OsPUB3* (**Figure 6D**). The transcript levels of *OsPUB2* and *OsPUB3* were slightly increased (1.1–1.5 times), in *Ubi:RNAi-OsPUB3* and *Ubi:RNAi-OsPUB2* plants, respectively (**Figure 6D**). These overexpressing and *RNAi*-mediated knock-down transgenic rice plants were used for phenotypic analysis in response to cold stress.

### *OsPUB2*- and *OsPUB3*-Overexpressing Transgenic Rice Plants Exhibited Markedly Enhanced Tolerance to Cold Stress Compared to Wild-Type Plants

**FIGURE 7:**
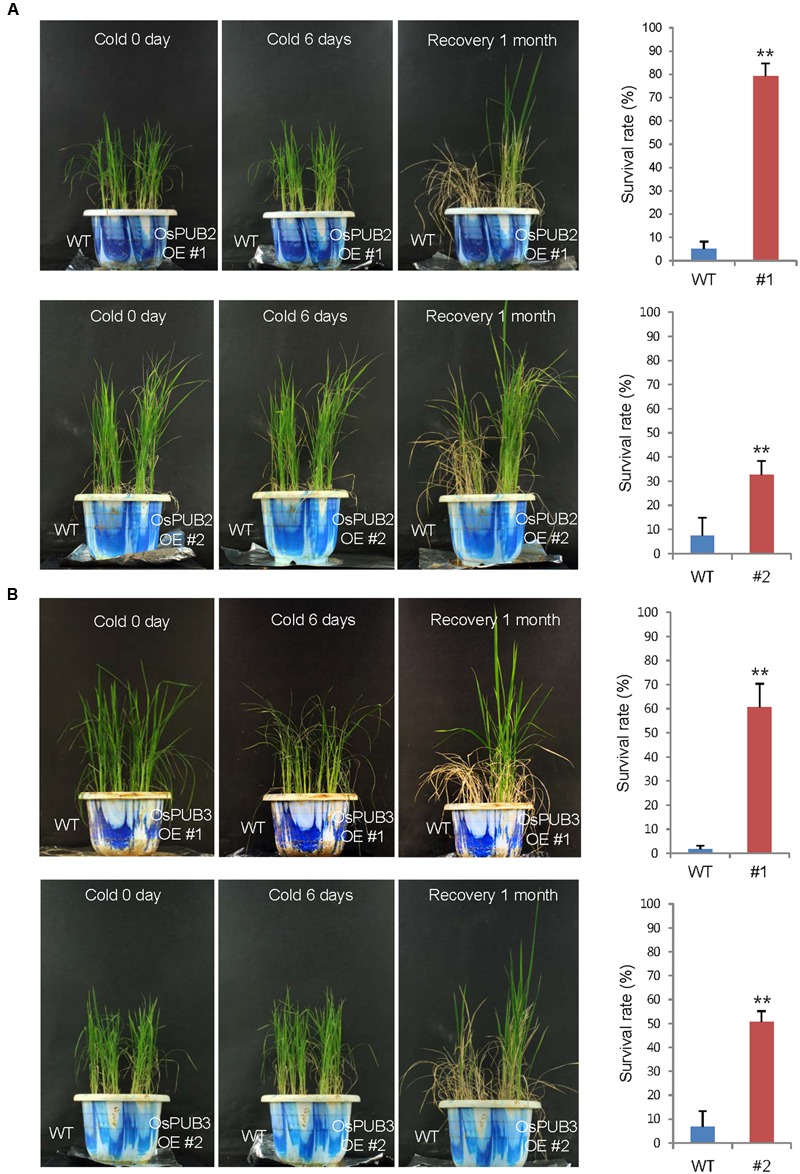
**Cold stress tolerant phenotypes of *OsPUB2*- and *OsPUB3*-overexpressing transgenic rice plants.** Overexpression of *OsPUB2* **(A)** and *OsPUB3* **(B)** conferred enhanced tolerance to cold stress compared to that in wild-type rice plants. Wild-type and T4 *Ubi:sGFP-OsPUB2* (lines #1 and #2) and *Ubi:sGFP-OsPUB3* (lines #1 and #2) transgenic rice plants were grown for 5 weeks under normal condition (28°C). These plants were transferred to cold room at 4°C for 6 days and recovered at 28°C. The survival rates of cold-treated plants were monitored. Data represent mean ± SE (*n* ≥ 5 independent experiments; more than 100 plants were used in each assay, ^∗∗^*P* < 0.01, Student’s *t*-test). OE, overexpressing transgenic plants.

Wild-type, T4 *Ubi:sGFP-OsPUB2* (lines #1 and #2), and T4 *Ubi:sGFP-OsPUB3* (lines #1 and #2) rice plants were grown at 28°C under long day (16-h light and 8-h dark) conditions for 5 weeks. These plants were then subjected to cold stress by transferring them to a cold room at 4°C. After 6 days of low temperature treatment, plants were retransferred to the growth room at 28°C, and their growth patterns were monitored. As shown in **Figure 7**, most of the wild-type plants exhibited pale green and yellowish leaves after cold stress. They were unable to grow and eventually died (survival rate = 1.7 ± 1.4–7.4 ± 6.4%). In contrast, *OsPUB2*- and *OsPUB3*-overexpressing plants showed markedly increased tolerance to cold temperature compared to the wild-type rice plant. The survival rates of *Ubi:sGFP-OsPUB2* was 79.1 ± 5.7% (line #1) and 32.5 ± 6.0% (line #2), whereas those of *Ubi:sGFP-OsPUB3* were 60.7 ± 9.7% (line #1) and 50.7 ± 4.4% (line #2) (**Figure 7**).

**FIGURE 8:**
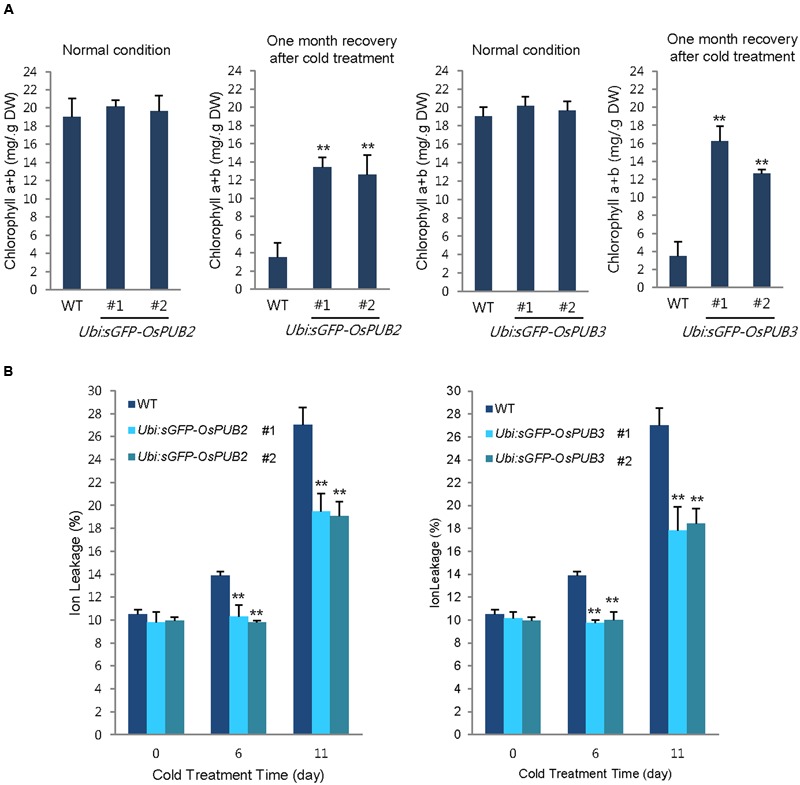
**Effects of *OsPUB2* and *OsPUB3* overexpression on leaf chlorophyll content and electrolyte leakage in response to cold stress. (A)** Total chlorophyll content of wild-type and T4 *Ubi:sGFP-OsPUB2* (lines #1 and #2) and *Ubi:sGFP-OsPUB3* (lines #1 and #2) transgenic rice plants. Light-grown, 5-week-old wild-type and transgenic plants were grown for 6 days under normal (28°C) or cold (4°C) condition. The amount of total leaf chlorophyll (chlorophyll a + chlorophyll b) was determined at normal growth condition and 1 month recovery after cold (4°C) treatment. Data indicate the mean ± SE (9 ≥ n ≥ 3 independent experiments; 10 plants were used in each experiment, ^∗∗^*P* < 0.01, Student’s *t*-test). **(B)** Electrolyte leakage analysis was performed using 8-day-old seedlings of wild-type and T4 *Ubi:sGFP-OsPUB2* (lines #1 and #2) and *Ubi:sGFP-OsPUB3* (lines #1 and #2) plants before and after cold (4°C) treatment in different time points (0, 6, and 11 days). Data represent ± SE (8 ≥ n ≥ 3 independent experiments; three plants were used in each experiment, ^∗∗^*P* < 0.01, Student’s *t*-test).

In addition, 5-week-old wild-type and *OsPUB2*/*OsPUB3-*overexpressing plants exhibited similar leaf chlorophyll content (chlorophyll a + chlorophyll b) under normal condition (**Figure 8A**). Consistent with their tolerance, *OsPUB2-* and *OsPUB3-*overexpressing progeny contained higher amount of chlorophyll compared to that in the wild-type plants in response to cold stress. At 1 month recovery of cold treatment (4°C), the leaf chlorophyll content of wild-type plants was 3.5 ± 1.6 mg/g DW, whereas those of *Ubi:sGFP-OsPUB2* and *Ubi:sGFP-OsPUB3* was 12.5 ± 2.2–13.5 ± 1.1 mg/g DW and 12.6 ± 0.5–16.2 ± 1.7 mg/g DW, respectively (**Figure 8A**). As a next experiment, electrolyte leakage from 8-day-old cold-stressed seedlings was determined. Wild-type and *OsPUB2/OsPUB3*-overexpressing plants were grown at 4°C for 0, 6, and 11 days, and whole seedlings were soaked in distilled water for measuring the rates of electrolyte leakage. The data showed that both *Ubi:sGFP-OsPUB2* and *Ubi:sGFP-OsPUB3* seedlings showed lower ion leakage (9.7 ± 0.2–10.3 ± 1.0% at 6 days and 17.8 ± 2.1–19.5 ± 1.5% at 11 days) than the wild-type (13.9 ± 0.4% at 6 days and 27.0 ± 1.5% at 11 days) plants in response to low temperature (**Figure 8B**). Overall, phenotypic analyses in **Figures 7** and **8** indicated that *OsPUB2*- and *OsPUB3*-overexpressors were more tolerant to severe cold stress than the wild-type plants, suggesting that OsPUB2 and OsPUB3 have positive roles in cold stress response in rice plants.

Next, we examined the phenotype of *Ubi:RNAi-OsPUB2* (lines #1 and #2) and *Ubi:RNAi-OsPUB3* (lines #1 and #2) knock-down transgenic lines. Unlike the overexpressing lines, *RNAi*-knock-down plants were very similar to wild-type plants in terms of tolerance to cold temperature (Supplementary Figure S5). The total chlorophyll content and electrolyte leakage rate of *RNAi*-knock-down plants were also indistinguishable from those of the wild-type rice plants (Supplementary Figure S6). These results led us to hypothesize that the basal levels of OsPUB2 and OsPUB3 might be considerably high, and hence, their partial suppression resulted in undetectable effects on the *Ubi:RNAi-OsPUB2* and *Ubi:RNAi-OsPUB3* knock-down transgenic lines. Alternatively, the suppression of *OsPUB2* could be complemented by *OsPUB3* and, conversely, the knock-down of *OsPUB3* could be rescued by *OsPUB2*. This assumption seems to be reasonable, since OsPUB2 and OsPUB3 shared high degree of sequence identity (75%), had a similar structure (**Figure 1A**), and formed a hetero-dimeric complex (**Figure 4**).

### Cold Stress-Induced Genes Are Up-Regulated in *Ubi:sGFP-OsPUB2* and *Ubi:sGFP-OsPUB3* Transgenic Plants Relative to That in the Wild-Type Plants

**FIGURE 9:**
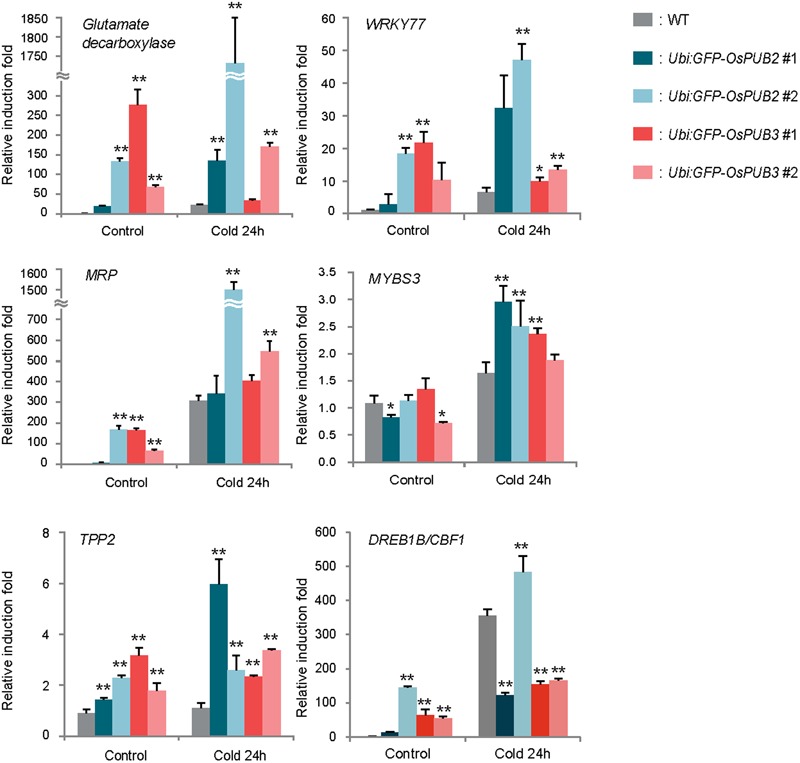
**Expression analysis of cold stress-inducible genes in wild-type and *OsPUB2*/*OsPUB3*-overexpressing transgenic rice plants.** Light-grown, 8-day-old wild-type and T4 *Ubi:sGFP-OsPUB2* and *Ubi:sGFP-OsPUB3* transgenic plants were subjected to cold (4°C) stress for 24 h. Induction patterns of five different cold-responsive genes were analyzed by real-time qRT-PCR by using the gene-specific primers listed in Supplementary Table S1. Data represent the fold inductions of *GAD, WRKY77, MRP4, MYBS3, TPP2*, and *DREB1B/CBF1* in response to cold stress relative to those in the control treatment. The relative expression level of each gene was normalized to that of *OsActin*, an internal reference gene. Data indicate mean ± SE (^∗^*P* < 0.05, ^∗∗^*P* < 0.01, Student’s *t*-test) from three independent experiments.

Because the ectopic expression of *OsPUB2* and *OsPUB3* conferred increased tolerance to cold stress in rice plants, we next determined whether OsPUB2 or OsPUB3 affected the expression profiles of cold-stress responsive genes. Light-grown, 8-day-old wild-type, *Ubi:sGFP-OsPUB2*, and *Ubi:sGFP-OsPUB3* transgenic seedlings were subjected to cold stress (4°C) for 24 h. Total RNA was isolated from the shoot tissue and analyzed using real-time qRT-PCR by using gene-specific primer sets (Supplementary Table S1). The expressions of *GAD* (Os03g13300; *glutamate decarboxylase*), *WRKY77* (Os01g40260), and *MRP4* (Os01g50100; *multidrug resistance protein 4*), which are cold stress-inducible genes (Jain et al., 2007; Su et al., 2010), were markedly upregulated in *Ubi:sGFP-OsPUB2* and *Ubi:sGFP-OsPUB3* transgenic plants than in the wild-type plants under both normal and cold-stressed conditions (**Figure 9**). Although, *TPP2* (Os10g40550; *trehalose-6-phosphate phosphatase* 2) was not induced by cold treatment in the wild-type rice plant, it was 2–6-fold upregulated in *OsPUB2/3*-overexpressing transgenic plants relative to wild-type plants after 24 h of cold treatment. The transcript level of *MYBS3* (Os01g50100) was similar in wild-type and *OsPUB2/OsPUB3*-overexpressing plants under normal condition, but was higher in the overexpressors after cold treatment (**Figure 9**). Expression of *DREB1B/CBF1* (CCAAT-binding factor) was higher in *Ubi:sGFP-OsPUB2/3* plants in normal condition relative to that of wild-type plant, but it became lower after cold treatment. These results suggested that the cold stress-tolerant phenotypes of *Ubi:sGFP-OsPUB2* and *Ubi:sGFP-OsPUB3* plants were correlated with increased expression levels of cold stress-related genes before or after low temperature treatment.

## Discussion

Rice U-box E3 Ub ligases are implicated in biotic stress responses such as innate immunity and PAMP-triggered cell death (Zeng et al., 2004; Ishikawa et al., 2014; Liu et al., 2015; Wang et al., 2015). Recent genome-wide expression analysis showed that *OsPUBs*, which contain the ARM repeat motif, are induced by a broad spectrum of abiotic stress, suggesting their roles in the response to environmental stimuli in rice plants (Sharma et al., 2014). Nevertheless, cellular roles of rice OsPUBs in response to abiotic stress are only beginning to be understood. In this study, we conducted functional analyses of two homologous rice U-box E3 Ub ligases, OsPUB2 and OsPUB3 (**Figure 1A**; Supplementary Figure S1A). Although, the transcripts of *OsPUB2* and *OsPUB3* were detected in various tissues of developing rice plants (**Figure 1B**), their expression profiles were different: *OsPUB2* was up-regulated by abiotic stresses, including low temperature, drought, and high salinity, whereas *OsPUB3* was constitutively expressed (**Figures 1C,D**). Consistent with their close overall sequence identity (75%) and conserved functional motifs, such as the U-box domain and ARM repeats, OsPUB2 and OsPUB3 showed an *in vitro* E3 Ub ligase activity (**Figure 2**) and similar subcellular localization patterns in the EXPO-like punctate bodies (**Figure 3**). Considering these features of OsPUB2 and OsPUB3, we speculated that they might play a cellular role in an inter-connected manner. This view was further supported by the results that OsPUB2 and OsPUB3 form a hetero-dimeric complex in addition to homo-dimers in yeast cells and *in vitro* (**Figure 4**).

**FIGURE 10:**
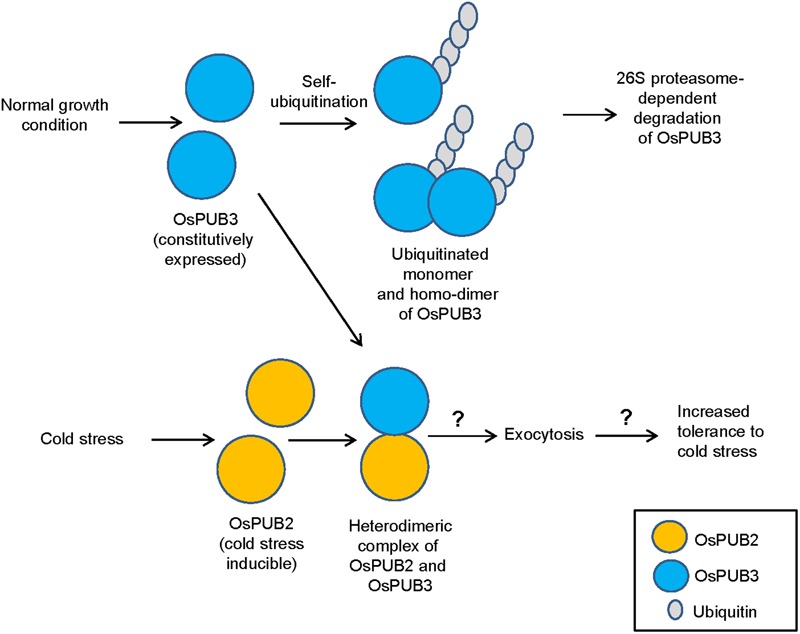
**A simplified working model of EXPO-localized OsPUB2 and OsPUB3 in response to cold stress in rice plants.** Under the normal growth conditions, constitutively expressed OsPUB3 is self-ubiquitinated and rapidly degraded. OsPUB2 is induced by cold stress and interacts with OsPUB3, which results in the formation of a more stable hetero-dimer. Hetero-dimeric complex of OsPUB2/OsPUB3 plays a positive role in the cold stress tolerance mechanism in rice plants. Possible involvement of OsPUB2/OsPUB3 hetero-dimeric form in the exocytosis process remains to be investigated.

E3 Ub ligases can catalyze their own ubiquitination, thereby targeting themselves in a form of negative feedback loop (Ryan et al., 2006; de Bie and Ciechanover, 2011). Both OsPUB2 and OsPUB3 exhibited self-ubiquitination activities *in vitro* (**Figure 2**) and were rapidly degraded in the cell-free extracts prepared from developing rice seedlings; they had apparent half-lives of 150–160 min (**Figure 5**). This rapid degradation of OsPUB2/OsPUB3 was delayed when the crude extracts of cold-treated seedlings were used (apparent half-lives of 200–280 min). Thus, the stability of OsPUB2/OsPUB3 might be regulated by low temperature stress. Moreover, a hetero-dimeric form of OsPUB2/OsPUB3 was more stable than homo-dimers in a cell-free degradation system (apparent half-lives of 250–350 min). These results are in agreement with the notion that OsPUB2 and OsPUB3 function coordinately in response to cold stress. Notably, the stability of OsPUB3 was more evidently increased than that of OsPUB2 in the cold-treated cell-free extracts and when they formed a hetero-dimer (**Figure 5**). *OsPUB3* was constitutively expressed in all the tissues examined in rice plants, whereas the basal level of *OsPUB2* was low, but rapidly increased by cold stress (**Figure 1**). With these results in mind, we hypothesized that (1) under the normal growth conditions, constitutively expressed OsPUB3 was self-ubiquitinated and rapidly degraded; (2) in response to cold stress, OsPUB2 was induced and interacted with OsPUB3, which resulted in the formation of a more stable hetero-dimer as well as homo-dimers; and (3) homo- and hetero-dimeric complexes of OsPUB2/OsPUB3 play a positive role in the cold stress tolerance mechanism in rice plants (**Figure 10**). In this scenario, however, it remains to be clarified how the OsPUB2/OsPUB3 hetero-dimeric form is more resistant to self-ubiquitination and subsequent degradation. Possible involvement of EXPO-localized OsPUB2/OsPUB3 in the exocytosis process should also be investigated.

Transgenic rice plants, in which *OsPUB2* and *OsPUB3* were over-expressed (*Ubi:sGFP-OsPUB2* and *Ubi:sGFP-OsPUB3*) or suppressed (*Ubi:RNAi-OsPUB2* and *Ubi:RNAi-OsPUB3*), were morphologically normal (**Figure 7**; Supplementary Figure S5). Their development and seed yields were indistinguishable from those of wild-type rice plants. Thus, OsPUB2 and OsPUB3 might not be involved in the normal cellular processes, but could specifically participate in the response to abiotic stress. Our results showed that *OsPUB2*/*OsPUB3*-overexpressors were markedly tolerant to low temperature treatment (4°C) with regard to survival rates (**Figure 7**), chlorophyll content and ion leakage (**Figure 8**), and expression levels of cold stress-inducible marker genes (**Figure 9**) compared to that in wild-type rice plants. These results strongly suggested that OsPUB2 and OsPUB3 are positive factors in response to cold stress. However, we failed to detect opposite phenotypes by using *Ubi:RNAi-OsPUB2* and *Ubi:RNAi-OsPUB3* knock-down transgenic plants (Supplementary Figures S5 and S6). These results could be reconciled by the interpretation that partial suppression of individual *OsPUB2* and *OsPUB3* was reciprocally complemented by *OsPUB3* and *OsPUB2*, respectively, since they play a role in an inter-connected or coordinated fashion. In addition, the basal levels of OsPUB2 and OsPUB3 might be sufficiently high; hence, their partial suppression resulted in undetectable effects on the *RNAi*-mediated knock-down transgenic lines. We obtained the T-DNA inserted loss-of-function knock-out mutant line (PFG_1B-02809.L) for *OsPUB2* from the rice T-DNA insertion sequence database^1^. However, this mutant was turned out to be an activation tagging line rather than a knock-out line. In addition, functional seeds of *ospub3* knock-out mutant line (PFG_K-04006.L) were unavailable. Identification and characterization of additional knock-out mutant lines will be necessary.

The Exo70 (exocyst component of 70 kDa) protein is one of the core components of an evolutionarily conserved exocyst vesicle-tethering complex (Chong et al., 2010). OsPUB2 and OsPUB3 are co-localized with the Exo70E2 subunit to EXPO-like cytosolic punctae (**Figure 3**). In *Arabidopsis*, an EXPO-localized U-box E3 Ub ligase PUB22 ubiquitinates Exo70B2 to regulate the PAMP-triggered responses (Stegmann et al., 2012). Most recently, Seo et al. (2016) reported that *Arabidopsis* PUB18 negatively regulates ABA-mediated stomatal movements by ubiquitinating Exo70B1. At this moment, however, it is unknown how EXPO-localized OsPUB2 and OsPUB3 regulate cold stress response in rice plants (**Figure 10**). Thus, the target proteins that are ubiquitinated by OsPUB2/OsPUB3 need to be identified to decipher the detailed modes of action of these rice U-box E3 Ub ligases. In addition to EXPO-like structure, OsPUB2 was found in the nuclei (**Figure 3**). This raised the possibility that OsPUB2 plays an additional role, unlike OsPUB3, which is predominantly present in the EXPO. In this respect, it should be noted that *OsPUB2*, in contrast to *OsPUB3*, is induced by not only low temperature but also drought and high salinity (**Figure 1C**). Thus, as a next experiment, we intend to perform phenotypic analysis to elucidate whether *OsPUB2*-overexpressors are tolerant to drought and salt stress.

## Conclusion

Our data indicate that two homologous U-box E3 Ub ligases OsPUB2 and OsPUB3 are the positive factors in response to low temperature stress in rice plants.

## Author Contributions

MB, LC, TO, Y-JJ, AL, and KP performed the experiments. MB, LC, BK, and WK analyzed the data. MB, LC, and WK designed the project and drafted the manuscript. WK supervised the project and complemented the writing.

## Conflict of Interest Statement

The authors declare that the research was conducted in the absence of any commercial or financial relationships that could be construed as a potential conflict of interest.

The reviewer CL and handling Editor declared their shared affiliation, and the handling Editor states that the process nevertheless met the standards of a fair and objective review.

## Footnotes

1 http://signal.salk.edu/cgi-bin/RiceGE
