# Supplementary material for: Homologous U-box E3 Ubiquitin Ligases OsPUB2 and OsPUB3 Are Involved in the Positive Regulation of Low Temperature Stress Response in Rice (Oryza sativa L.)
Source: Front Plant Sci. 2017 Jan 20;8:16. doi: 10.3389/fpls.2017.00016 (PMC5247461; doi:10.3389/fpls.2017.00016)
Supplement: Supplementary file 1 [file Table_1.DOCX]

| **SUPPLEMETAL TABLE S1. List of synthetic oligonucleotide sequences used in this study** | | |  |
| --- | --- | --- | --- |
| Oligonucleotide name | Sequence | Purpose |  |
| OsPUB2 CDS F | ATGGCCAACGCGAGGAACG | gene cloning |  |
| OsPUB2 CDS R | TCACGACGCTGTAATGGCCGTC | gene cloning |  |
| OsPUB2 CDS no stop R | CGACGCTGTAATGGCCGTC | Construction for transgenic plants lines |  |
| OsPUB3 CDS F | ATGGCTGGTAATCCGGCGG | gene cloning |  |
| OsPUB3 CDS R | TCACGAAGCCATCATGGCAG | gene cloning |  |
| OsPUB3 CDS no stop R | CGAAGCCATCATGGCAGGGGG | Construction for transgenic plants lines |  |
| OsPUB2^C281A^ F | GGACTTCCGGGCCCCCATCTCTC | Construction for mutant form of protein |  |
| OsPUB2^C281A^ R | GAGAGATGGGGGCCCGGAAGTCC | Construction for mutant form of protein |  |
| OsPUB3^C280A^ F | TTCCGGGCCCCAATCTCTCTCGAT | Construction for mutant form of protein |  |
| OsPUB3^C280A^ R | ATCGAGAGAGATTGGGGCCCGGAA | Construction for mutant form of protein |  |
| OsPUB2 RNAi F | GCACAACGAATGCTTGTACAA | Construction for transgenic plants lines |  |
| OsPUB2 RNAi R | GAGATGATCACAATTCACAAGCA | Construction for transgenic plants lines |  |
| OsPUB3 RNAi F | ATGGCTTCGTGAAAACCA | Construction for transgenic plants lines |  |
| OsPUB3 RNAi R | GTACAGAAATGTGCATGTG | Construction for transgenic plants lines |  |
| OsPUB2 RT F | TCCTCGACGCCAACAAGAAG | RT-PCR |  |
| OsPUB2 RT R | CATCAGCTCCCATATCGCCC | RT-PCR |  |
| OsPUB3 RT F | TGTCGAACAAGGCCCTCAAG | RT-PCR |  |
| OsPUB3 RT R | GAGCCGGACTTGGATAGCAG | RT-PCR |  |
| OsUbiquitin F | ATGCAGATCTTTGTGAAGACATTG | RT-PCR |  |
| OsUbiquitin R | TTACTGACCACCACGGAGGC | RT-PCR |  |
| Hph probe F | ATGAAAAAGCCTGAACTCACC | Southern blot probe |  |
| Hph probe R | CTATTCCTTTGCCCTCGG | Southern blot probe |  |
| OsAct qRT F | GCTGACCGTATGAGCAAGGA | qRT-PCR |  |
| OsAct qRT R | TGAACAATTGCTGGACCCG | qRT-PCR |  |
| MYBS3 F | CCTTTCTGGCAAAATCAGAAAGA | qRT-PCR |  |
| MYBS3 R | ATGAACTGGAACAGGCTTGACA | qRT-PCR |  |
| DREB1B F | AGCTCGCCGGCTCCGACA | qRT-PCR |  |
| DREB1B R | GGGAGAAATCTGGCACATTCC | qRT-PCR |  |
| TPP2 F | AGGATGCATTCAAGGTTCTGA | qRT-PCR |  |
| TPP2 R | CAAGATGCCAGTTTCTTCAGG | qRT-PCR |  |
| MRP4 F | CAGGCAGAGGAACAGGTGAT | qRT-PCR |  |
| MRP4 R | CGTACCGGAACAAGCTGAAC | qRT-PCR |  |
| GAD F | AAGACGCTGCTGATTGATATGAT | qRT-PCR |  |
| GAD R | TGGTAGCTCACACCATGAATGTA | qRT-PCR |  |
| WRKY77 F | GGAATGGACAATTAGTTTGTCTCC | qRT-PCR |  |
| WRKY77 R | ATATATCGATGGGCCGTAATTTT | qRT-PCR |  |
