# Supplementary material for: Homologous U-box E3 Ubiquitin Ligases OsPUB2 and OsPUB3 Are Involved in the Positive Regulation of Low Temperature Stress Response in Rice (Oryza sativa L.)
Source: Front Plant Sci. 2017 Jan 20;8:16. doi: 10.3389/fpls.2017.00016 (PMC5247461; doi:10.3389/fpls.2017.00016)
Supplement: Supplementary file 2 [file Presentation_1.PDF]

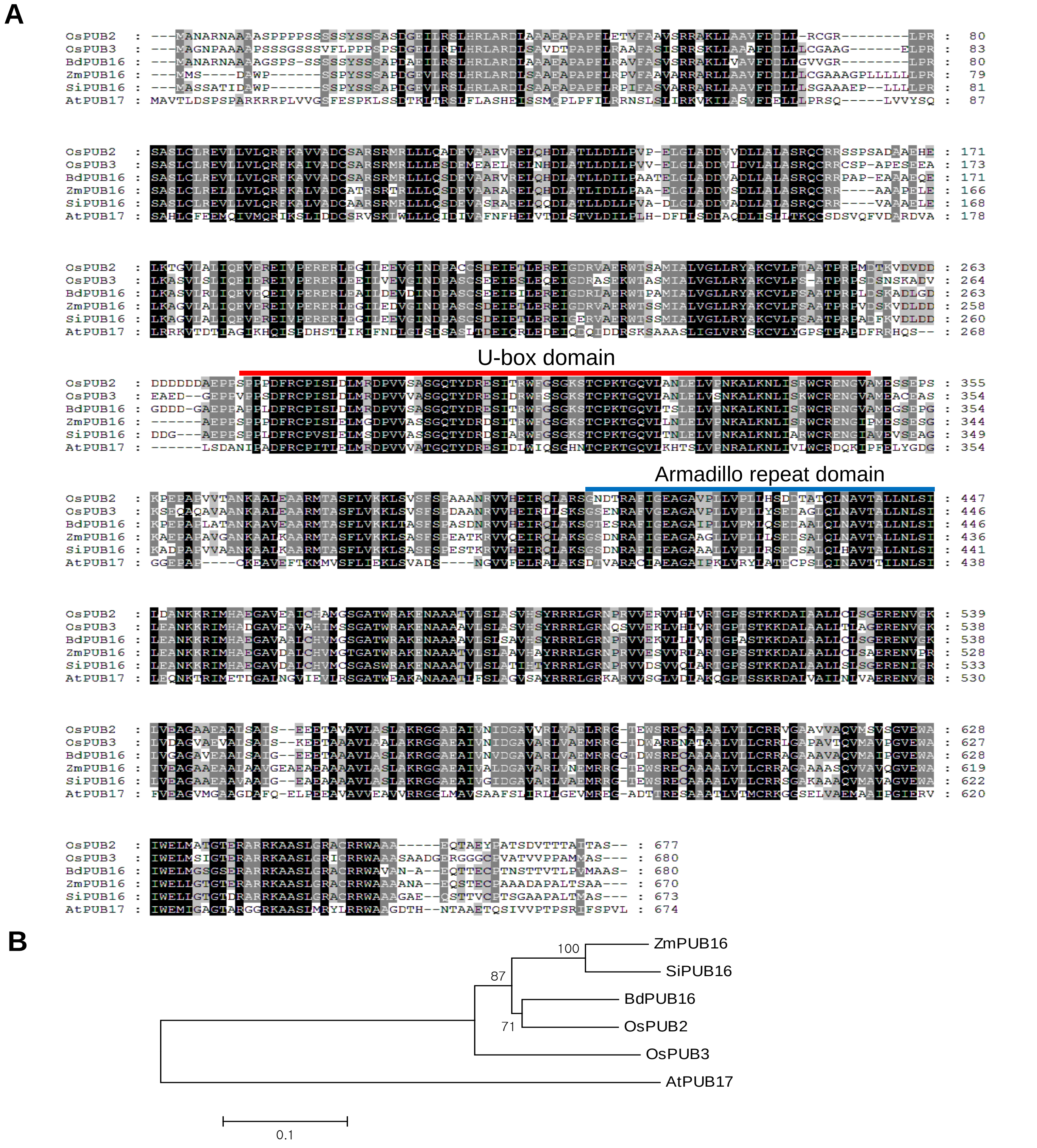

SUPPLEMENTARY FIGURE S1 | Sequence analysis of rice OsPUB2 and OsPUB3. (A) Comparison of the deduced amino acid sequences of OsPUB2 (XP_015637531) and OsPUB3 (XP_015621922) with homologous proteins from millet (*Setaria italica*) (SiPUB16; XP_004961754), false brome (*Brachypodium distachyon*) (BdPUB16; XP_003566140), maize (*Zea mays*) (ZmPUB16; XP_008649757), and *Arabidopsis* (AtPUB17; NP_195803). Amino acids identical in all six proteins are highlighted in black. Amino acid residues conserved in at least five of the six sequences are shaded. The U-box and ARM domains are indicated by solid lines. **(B)** Phylogenetic relationships of OsPUB2 and OsPUB3 with putative PUBs from rice, millet, false brome, maize, and *Arabidopsis*. The tree was constructed using MEGA4 software with the neighbor-joining method.

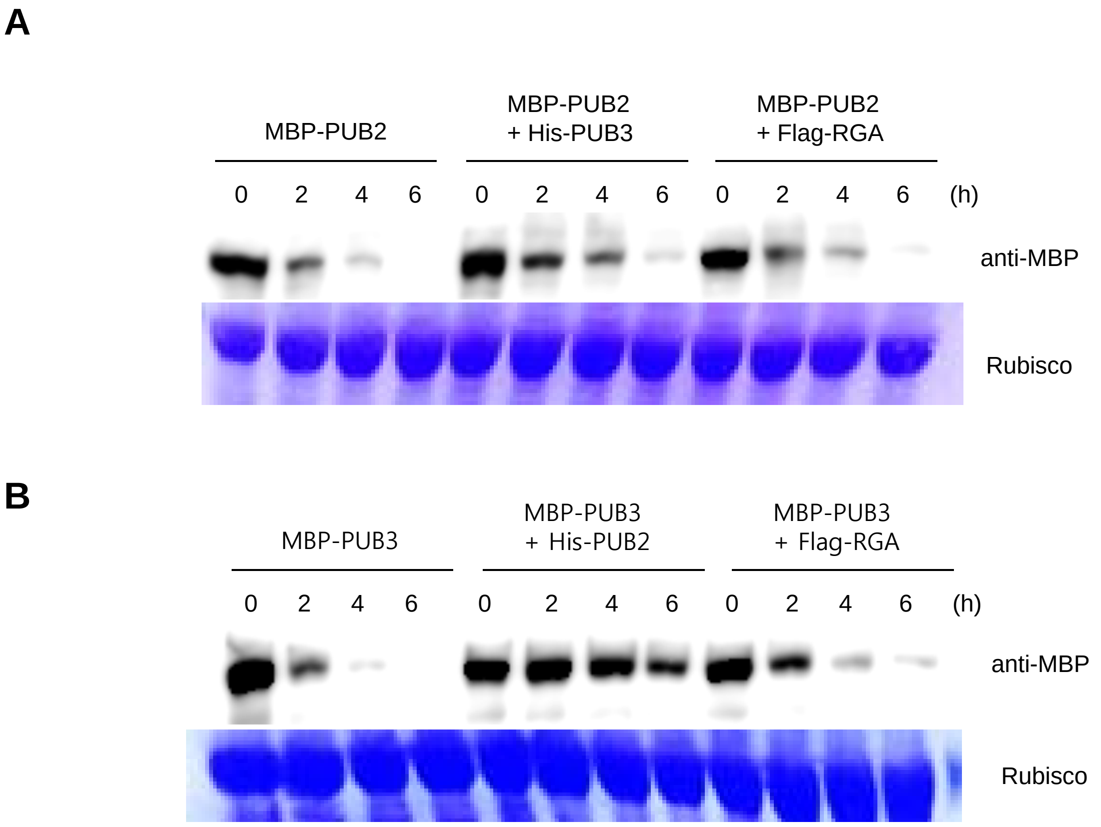

SUPPLEMENTARY FIGURE S2 | In vitro cell-free degradation assay of homo- and hetero-dimeric complexes of OsPUB2 (A) and OsPUB3 (B) in the presence of RGA. The MBP-OsPUB2 and MBP-OsPUB3 were incubated for different time periods (0, 2, 4, and 6 h) in the presence or absence of $(His)_6$-OsPUB3, $(His)_6$-OsPUB2, and Flag-RGA, respectively, with mock-treated crude extracts. The levels of proteins were detected by immuno-blotting with anti-MBP antibody. Rubisco was used as a loading control.

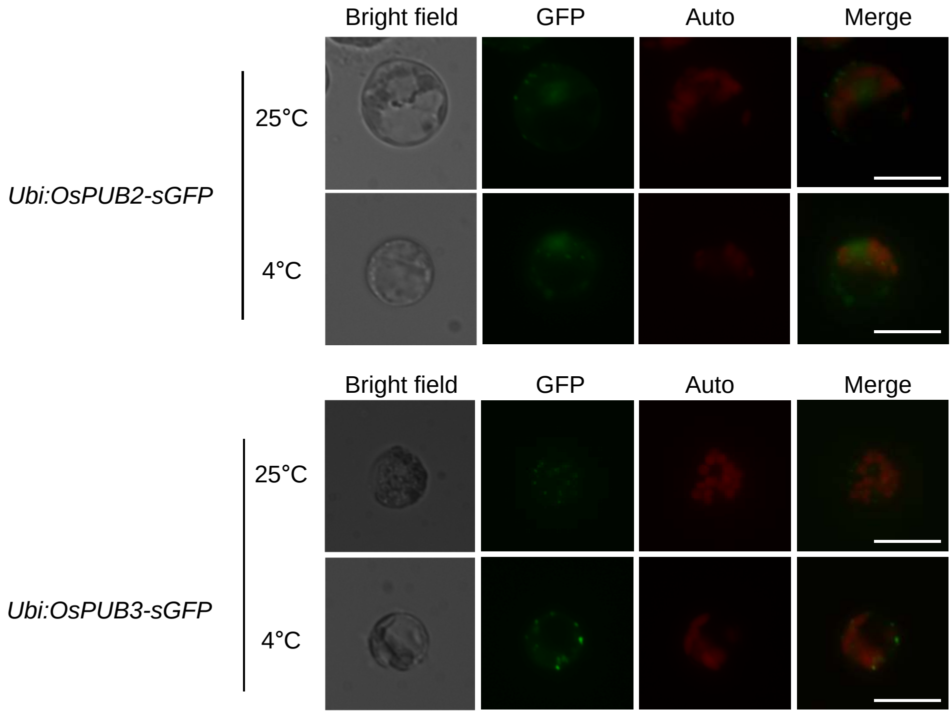

SUPPLEMENTARY FIGURE S3 | Subcellular localization of OsPUB2 and OsPUB3 before and after cold treatment in transgenic rice protoplasts. Protoplasts were prepared from 11-day-old seedlings from *Ubi:sGFP-OsPUB2* and *Ubi:sGFP-OsPUB3* transgenic rice plants before and after cold treatment for 48 h in the presence of MG132 (50 μM). Fluorescent signals were visualized by fluorescence microscopy. Bars = 5 μm.

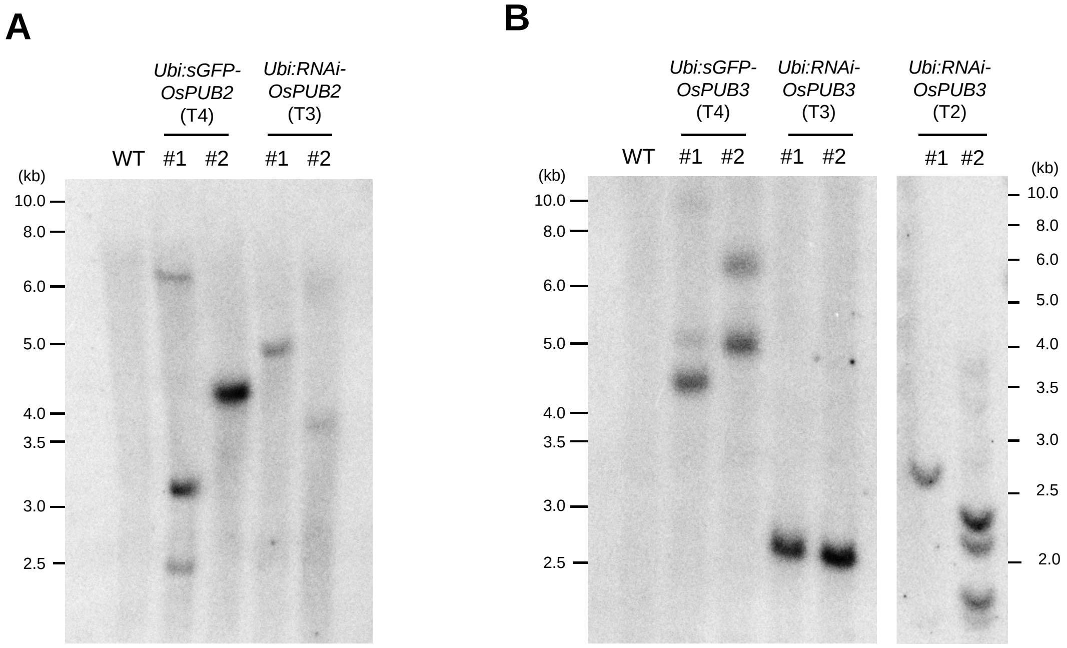

SUPPLEMENTARY FIGURE S4 | Genomic Southern blot analysis. (A) Total leaf genomic DNA was isolated from wild type (WT), T4 *Ubi:sGFP-OsPUB2,* and T3 *Ubi:RNAi-OsPUB2* rice plants. DNA was digested by *Bam*HI and hybridized with ^{32}P-labeled hygromycin B phosphotransferase (*Hph*) probe under high stringency conditions. **(B)** Total leaf genomic DNA was isolated from wild type (WT), T4 *OsPUB3*-overexpressing, and T3 and T2 *Ubi:RNAi-OsPUB3* rice plants. DNA was digested by *Eco*RI and hybridized with ^{32}P-labeled hygromycin B phosphotransferase (*Hph*) probe under high stringency conditions. Southern blot analysis of T2 *Ubi:RNAi-OsPUB3* rice plant (right panel) is also presented to confirm that *Ubi:RNAi-OsPUB3* transgenic lines #1 and #2 are independent.

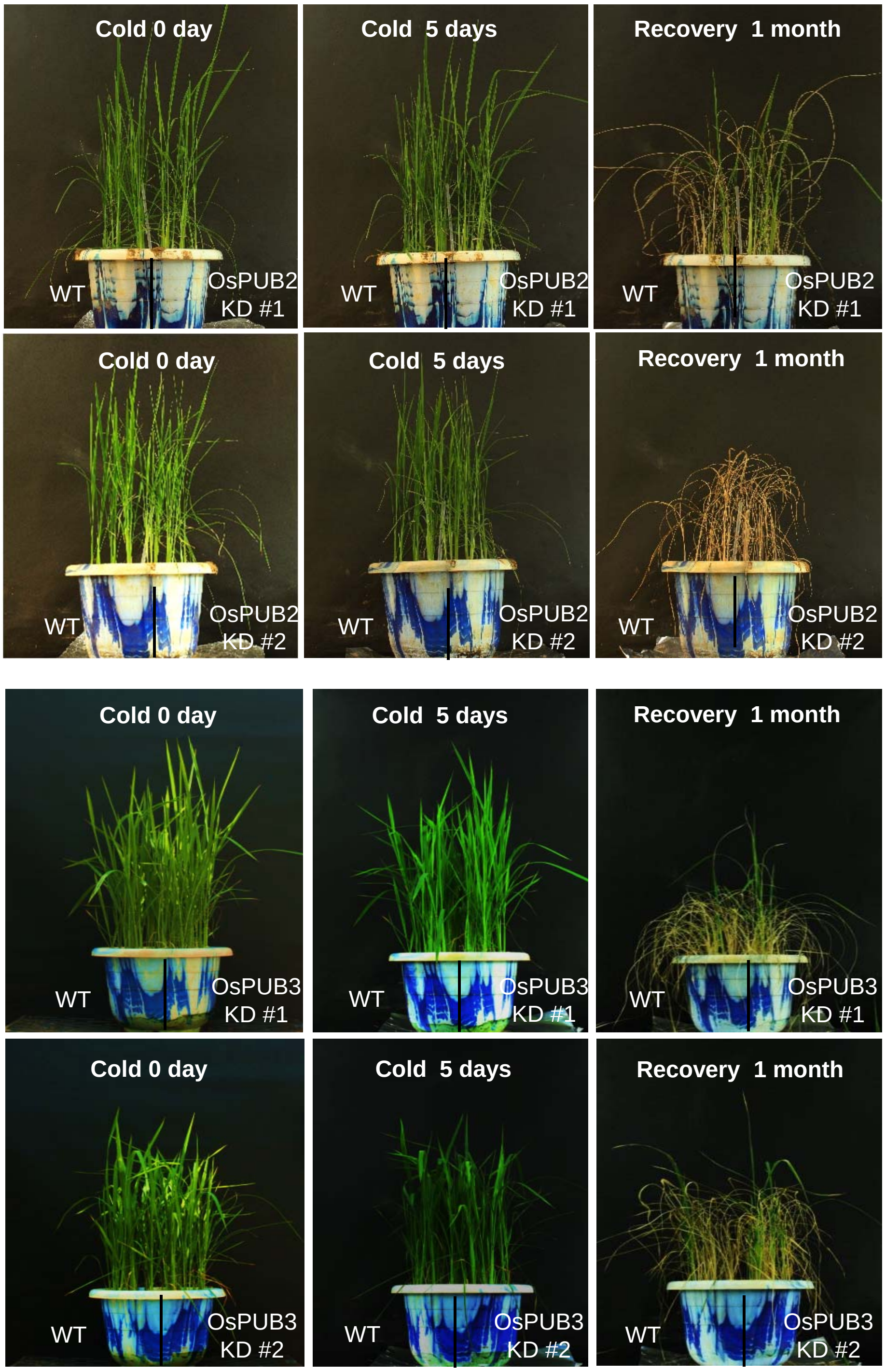

SUPPLEMETARY FIGURE S5 | Cold phenotypes of *OsPUB2* and *OsPUB3* RNAi knock-down transgenic rice plants. Partial suppression of *OsPUB2* and *OsPUB3* resulted in the similar cold tolerance phenotypes as compared to wild type rice plants. Wild type and T3 *Ubi:RNAi-OsPUB2* (independent lines #1 and #2) and *Ubi:RNAi-OsPUB3* (lines #1 and #2) transgenic rice plants were grown for 5 weeks under normal condition (28°C). These plants were then transferred to cold room at 4°C for 5 days and recovered at 28°C. Data represents means ± SE (n ≥ 3 independent experiments; more than 100 plants were used in each assay). KD, RNAi-mediated knock-down.

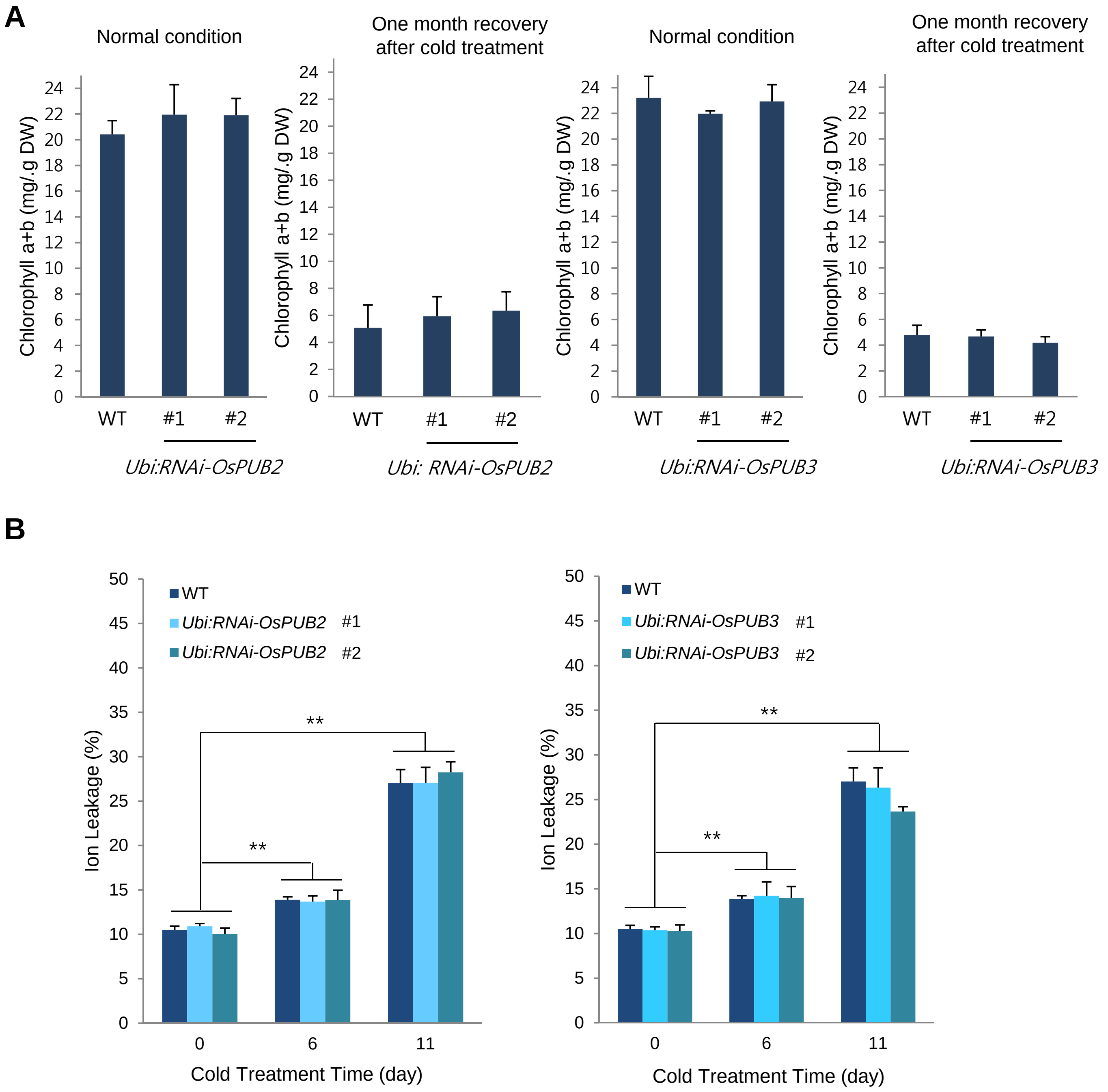

SUPPLEMETARY FIGURE S6 | Effects of *OsPUB2* and *OsPUB3* suppression on chlorophyll content and electrolyte leakage in response to cold stress. (A) Total chlorophyll content of wild type and T3 *Ubi:RNAi-OsPUB2* (lines #1 and #2) and *Ubi:RNAi-OsPUB3* (lines #1 and #2) transgenic rice plants. Light-grown, 5-week-old wild type and transgenic rice plants were grown for 6 days under normal (28°C) or cold (4°C) condition. The amount of total leaf chlorophyll (chlorophyll a + chlorophyll b) was estimated at normal growth condition and one month recovery after cold (4°C) treatment. Data indicate the means ± SE (9≥ n ≥ 4 independent experiments; 10 plants were used in each experiment) **(B)** Electrolyte leakage analysis was performed using 8-day-old seedlings of wild type and T3 *Ubi:RNAi-OsPUB2* (lines #1 and #2) and *Ubi:RNAi-OsPUB3* (lines #1 and #2) plants before and after cold (4°C) treatment (0, 6, and 11 days). Data represent means ± SE (8 ≥ n ≥ 3 independent experiments; 3 plants were used in each experiment, **P<0.01, Student's *t*-test).
